# The Core Matters: Aggregation of Neutral Ligand-Coated Nanoparticles in Membranes

**DOI:** 10.1021/acs.jpcb.6c03349

**Published:** 2026-09-01

**Authors:** Jahmal J. Ennis, Julianne C. Griepenburg, Grace Brannigan

**Affiliations:** † Center for Computational and Integrative Biology, 15636 Rutgers University-Camden, Camden, New Jersey 08012, United States; ‡ Department of Physics, Rutgers University-Camden, Camden, New Jersey 08012, United States

## Abstract

The aggregation of gold nanoparticles (AuNPs) influences their optical properties, including localized surface plasmon resonance, through plasmonic coupling between adjacent particles. For hydrophobic AuNPs embedded in lipid membranes, however, the mechanisms governing aggregation remain poorly understood. Although coarse-grained molecular dynamics simulations are well suited to study these processes, commonly used ligand–nanoparticle (NP) models neglect explicit core interactions. Here, we test whether core properties regulate NP aggregation by simulating multiple Martini NP models in a membrane environment. In contrast to the assumption that surface ligands effectively shield the core, we find that interactions with the environment remain sensitive to core parameters even at maximum ligand coverage. Furthermore, core–solvent interactions are consistent with a mechanism that directly drives nanoparticle aggregation. These results suggest that core interactions can contribute to the aggregation of functionalized nanoparticles.

## Graphical abstract

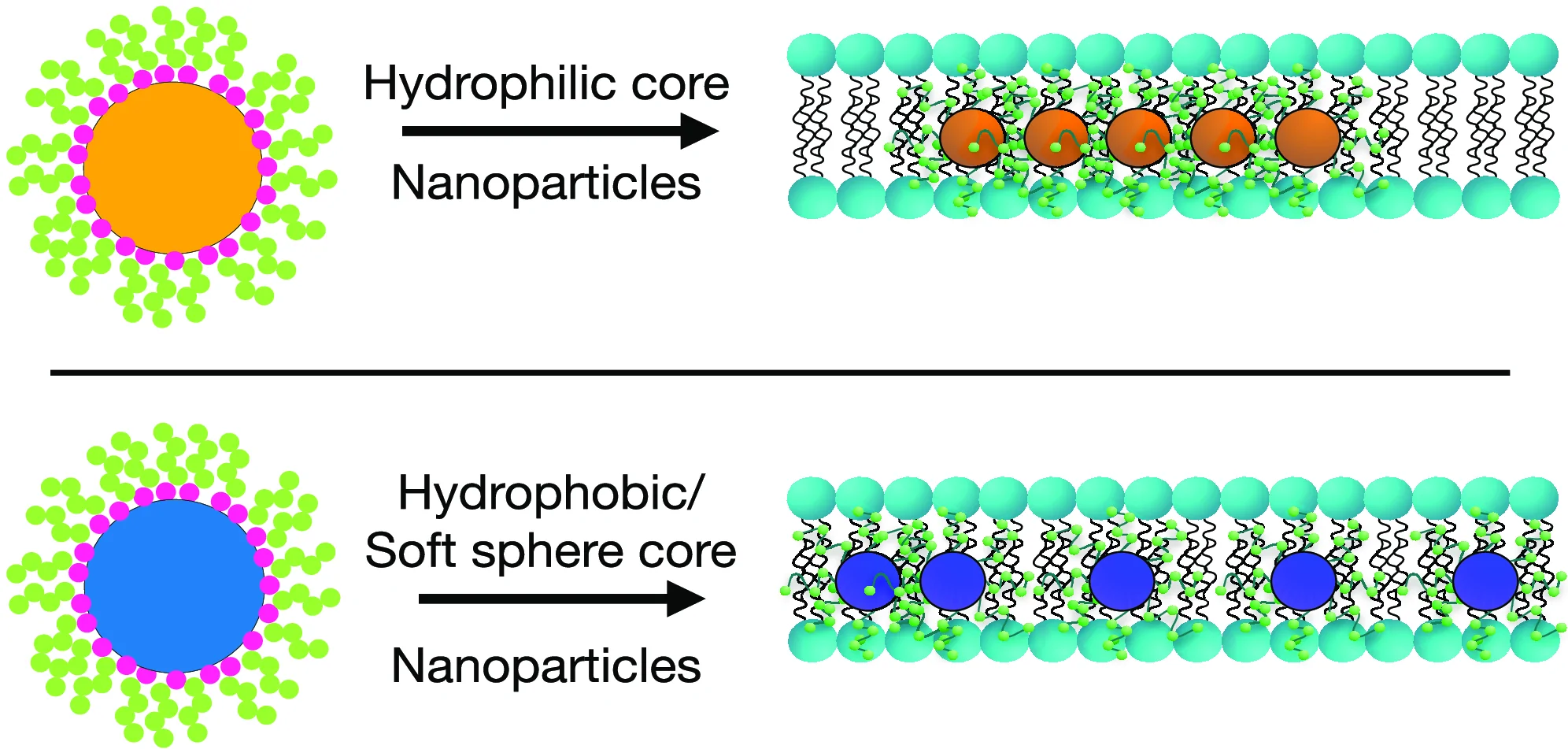

## Introduction

Gold nanoparticles (AuNPs) are increasingly prevalent in biomedical applications such as drug delivery, nanomedicine, cellular imaging, tumor treatment, and photothermal cancer therapy^1–6^ due to their tunable surface and optical properties. These optical properties originate from localized surface plasmon resonance (LSPR), the incident light-driven oscillation of conduction electrons in the nanoparticle.^7^ Subsequent relaxation transfers energy to the surrounding environment, producing a thermomechanical response^8–10^ that can be exploited in noninvasive, light-based medical applications.^11^ For instance, in plasmonic nanocarriers—light responsive vesicular systems designed for drug delivery—AuNPs are functionalized with hydrophobic ligands to embed within bilayer membranes and serve as photosensitizers to induce membrane disruption.^12^ Through this mechanism, AuNPs have the potential to act as nanoscale transducers, providing spatial and temporal control over optical energy transfer to the surrounding environment.

In biomedical applications, precise control of AuNP optical properties is more challenging due to unpredictable aggregation, which can significantly alter the optical response. Cryo-electron microscopy shows that hydrophobic AuNPs embedded in lipid membranes spontaneously aggregate,^13–16^ which can enhance two-photon absorption for deep-tissue imaging^17,18^ but often shifts localized surface plasmon resonance and induces off-resonant photoexcitation.^19^ In aqueous environments, AuNP aggregation is commonly controlled by surface functionalization with charged ligands, such as citrate anions, which impart a negative surface charge and reduce aggregation.^20,21^ In contrast, strategies for controlling AuNP aggregation in lipid membranes remain limited, with the only successful approach relying on bulky molecules tethered to the AuNPs to increase steric hindrance.^22^ These complex and bulky modifications can alter nanoparticle–membrane interactions; thus small, simple functional groups are optimal for biomedical applications. However, there is a lack of mechanism-driven strategies for controlling membrane-mediated aggregation for AuNPs with simpler functional groups, such as alkanethiols.

Many theoretical models of AuNP aggregation employ analytical frameworks that approximate nanoparticles as unliganded hard spheres,^23^ thereby isolating membrane- or lipid-mediated contributions to aggregation. Within this context, two widely studied deformation-based mechanisms are membrane elastic deformation and lipid chain disordering, both of which predict that aggregation reduces the free-energy cost associated with membrane perturbations by reducing the nanoparticle–lipid interfacial area. In the elastic deformation framework, lipid membranes can be described as elastic continua capable of bending and shape deformation;^24–26^ nanoparticle insertion induces local curvature, and aggregation minimizes the total bending energy.^27–30^ In an alternative framework, nanoparticle insertion disrupts lipid order, leading to unfavorable packing frustration and increased attraction between AuNPs.^31–34^ While these models capture key thermodynamic drivers of aggregation, they do not explicitly account for nanoparticle surface chemistry, which could influence aggregation.

Hard sphere (hsNP) models often neglect explicit surface interactions and thus cannot distinguish the behavior of gold nanoparticles (AuNPs) from that of other nanoparticle types, despite fundamental differences that may influence aggregation. In particular, the gold core exhibits strong, specific interactions that differ markedly from those of typical organic nanoparticle cores. Gold surfaces interact strongly with water, structuring the interfacial water. This process leads to hydrophobic hydration, in which water molecules hydrogen-bond to a rigid surface, effectively rendering it hydrophobic.^35^ In addition, gold–gold interactions are strengthened by epitaxial forces, resulting in elevated surface energies and enhanced reactivity at the nanoscale.^36,37^ Such features are largely absent in purely hydrophobic nanoparticles, which interact weakly with polar molecules and possess low surface energies in aqueous solution^38^ compared to gold surfaces.^39^ AuNPs also exhibit heterogeneous surface structures comprising distinct facets, grooves, and lattice planes, which strongly influence the adsorption of organic molecules.^40^ In contrast, hydrophobic substrates, such as carbon nanoparticles, are smooth and uniform, thus limiting differential adsorption.^41^ These core-specific interactions may contribute to AuNP aggregation and produce behavior that cannot be captured by models developed for hsNPs. However, whether ligand shells sufficiently screen the core to suppress core interactions remains an open question that is inaccessible to mean-field analytical models.

Molecular dynamics simulations can be used to detect effective pairwise interactions in complex systems. Direct observation of AuNP aggregation at the atomistic level is impractical due to the limited diffusion within lipid membranes that can be observed over reasonable atomistic simulation time scales. Although several atomistic gold models compatible with biological force fields have been developed,^37^ overcoming kinetic limitations is achieved through coarse-grained (CG) nanoparticle (NP) models used in simulation studies motivated by gold nanoparticle applications.^33,34,42–47^ In particular, Martini CG NP models have been used to study cellular uptake,^42^ surface binding,^48^ and membrane insertion of nanoparticles.^49^ The Martini framework includes hydrophobic, hydrophilic, and charged beads, but no systematically parametrized metal bead. Thus, previous studies of gold nanoparticles employed ad hoc representations of the AuNP core, including hydrophobic (hNP, Figure 1),^42^ hydrophilic (pNP, Figure 1),^48^ and soft-sphere (sNP, Figure 1)^49^ models. If surface ligands fully shielded the core, these models would be expected to yield equivalent behaviors to each other or a gold bead. However, the maximum geometric ligand coverage (defined as the ratio of thiol groups to surface gold atoms) of an AuNP is only approximately 60%,^50,51^ indicating that core properties may still influence NP interactions.

**Figure 1:**
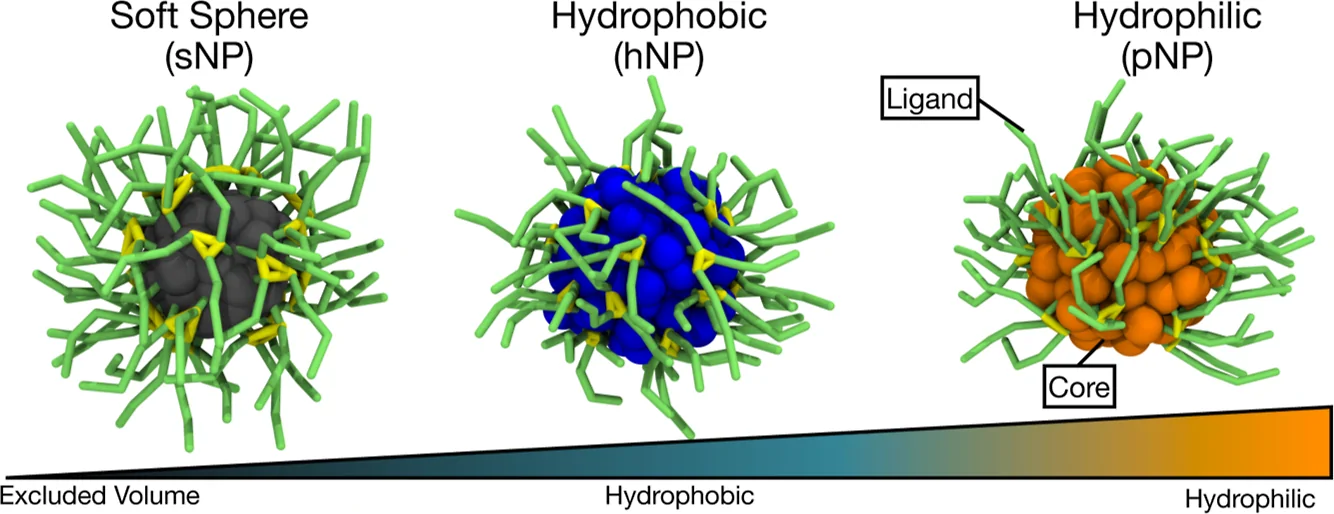
Nanoparticle models considered in this study. Nanoparticle cores are in orange, blue, or gray, and ligands are shown in yellow and green. Nanoparticle cores are modeled with different classes of bead types: hydrophilic p-type (P5–P1), hydrophobic c-type (C1–C5), or non-Martini repulsive s-type beads. Nanoparticle models are coated with hydrophobic alkanethiol ligands and are identified as follows: soft sphere nanoparticles (sNP) contain a purely repulsive s-type core, hydrophobic nanoparticles (hNP) have a c-type core, and hydrophilic nanoparticles (pNP) have p-type cores.

In this work, we test the assumption that NP aggregation in lipid membranes is independent of core properties. We investigate NP models within the Martini 2 force field because of its widespread popularity, reliability, and library of preparameterized biological molecules. The NP models were investigated within 1-palmitoyl-2-oleoyl-*sn*-glycero-3-phosphocholine membranes, to model experimental systems that demonstrate gold nanoparticle aggregation.^13^ First, we elucidate the mechanism of NP aggregation by investigating the interactions between NPs and their surrounding solvent environment, by decoupling ligand effects from core effects. Next, we characterize how the core model and ligands determine the aggregation state of NPs. As a result, we find that core interactions with the aqueous solvent can modulate long-range membrane-mediated interactions between NPs. Finally, we discuss the limitations of this study and the Martini 2 NP models in lipid environments. To our knowledge, this is the first computational investigation examining how ligand length influences the aggregation of lipid membrane-embedded nanoparticles.

## Methods

### NP Modeling

The initial atomistic AuNP structure was created from *charmm-gui nanomaterial modeler*.^52^ All gold nanoparticles have a 2 nm icosahedral core, 189 beads, and alkanethiol ligands attached to the gold surface by a gold–sulfur bond. Atomistic structures were coarse-grained using the gmx traj utility in GROMACS,^53^ guided by a custom index file defining the atom-to-bead mapping scheme. Coarse-grained bead positions were calculated from the center of mass of their corresponding atomic groups as shown in Figure 2. Four water molecules were mapped to one Martini bead, and lipids were mapped with an average of four heavy atoms per bead according to the traditional Martini mapping scheme.^54^ Gold and sulfur atoms were mapped 1:1, while the ligand atoms were mapped 4:1. The ligands and core were parametrized as in Tables S1 and S2, and Figure S40. The Martini beads used in this study were implemented with standard nonbonded potentials as defined in Marrink et al.,^54^ except for soft sphere (SS) beads. Within the Martini framework, P5 and P1 are polar, N0 is nonpolar, and C1 and C5 are hydrophobic. The water affinity for the beads decreases in the order P5 > P1 > N0 > C5 > C1. Soft sphere beads interacted with all other beads via an excluded volume, the attractive term of the Lennard-Jones potential for a P5 Martini bead, as described in eq S3. Due to the absence of AuNP-specific models in Martini 3, we utilized the Martini 2.2 framework for all nanoparticle implementations.

**Figure 2:**
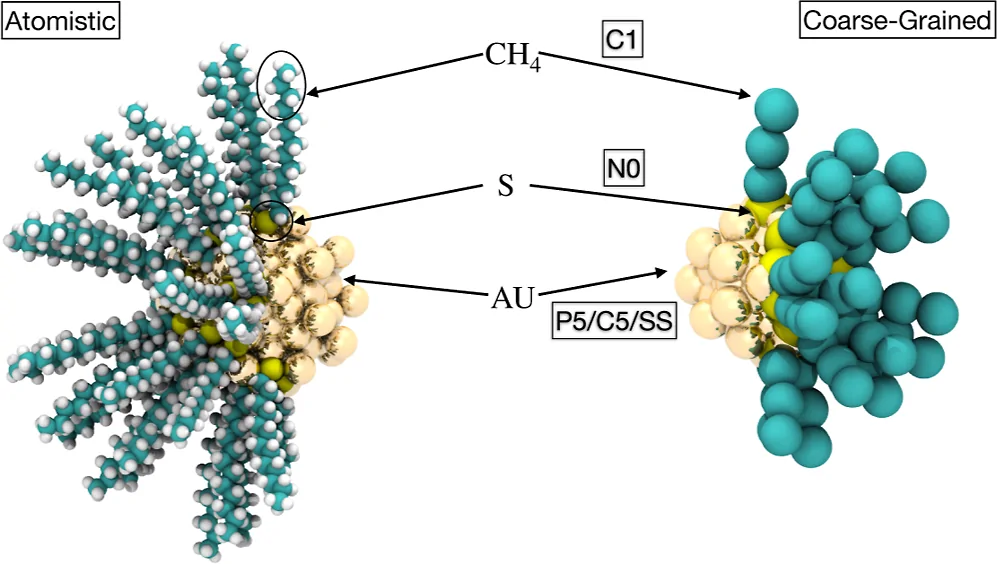
Atomistic and coarse-grained representations of nanoparticles. Only half of the ligands are shown for clarity. Au atoms were mapped 1:1 to either a P5, C5, SS bead for the dimerization and large scale aggregation studies or a P5, P1, N0, C1, C5 or SS bead for single nanoparticle studies. Sulfur was mapped 1:1 to an N0 bead, and carbons were mapped 4:1 to C1 beads.

Ligands are uniformly arranged around the core, covering approximately 60% of the NP surface, matching the atomistic models; coverage was not independently quantified postmapping. Ligand connectivity is shown in Figure S40. A harmonic restraint was applied to any pairs of core or N0 ligand beads that were separated by less than 5.9 Å in the initial structure; the force constant was 5000 kJ mol^–1^ nm^–2^ and the equilibrium distance for the pair was determined by the distance in the initial structure. All topology files, mapping scripts, and tutorials for building nanoparticles using this workflow are publicly available at https://github.com/BranniganLab/nanoparticle-aggregation-2026.

### System Setup

We added NPs to POPC membranes, solvated with water and NaCl using the standard insane^55^ procedure, then varied the number of NPs embedded (1, 2, or 10 NPs), and the ligand length (8, 12, 16, 20, or 24 carbons). Nanoparticles were embedded in different-sized membranes: single nanoparticles were embedded in 15 × 15 nm^2^ membranes, dimers in 25 × 25 nm^2^ membranes, and 10 nanoparticle systems in 45 × 45 nm^2^ membranes. Lipids within 2 nm of the NP were removed from the system using VMD^56^ to relieve steric clashes between lipids and ligands. Systems were subsequently energy-minimized and equilibrated in the *NPT* ensemble, during which any transient voids introduced by removing sterically clashing lipids fully closed. The chain order was unaffected, suggesting the membrane fluidity was unaffected by the nanoparticle embedding procedure, as determined by calculating the lipid tail order parameters as a function of radial distance from the nanoparticle (Figure S39).

### Simulation Details

Simulations were run using the Martini 2.2 force field^54^ in *gromacs* version 2016.1.^53^ We ran energy minimization for 10,000 steps for each system and production for 20 μs (single NP) or 5 μs (10 NPs) with a 0.025 ps time step. Simulations were run in the *NPT* ensemble with a pressure of 1.0 bar, using the Berendsen barostat, under semi-isotropic conditions with compressibility of 3.5 × 10^–5^ bar^–1^, pressure coupling time constant of 3.0 ps, temperature of 320 K, a velocity-rescaling thermostat,^57,58^ and a van der Waals cutoff of 1.2 nm.

### Analysis

#### Unbiased Simulation Analysis

All trajectories were visualized using VMD^56^ and all analysis was done over the last 10 or 2.5 μs of the simulation. Any 2 nanoparticles with a center of mass distance less than 2.5 nm were considered to be in the same aggregate. ⟨*F*_a_⟩ is the time-averaged ratio between total nanoparticles in the largest aggregate *N*_l_ and total nanoparticles in a system *N*_s_, $\left\langle F_{a}\right\rangle =\left\langle \frac{N_{l}}{N_{s}}\right\rangle$ . *F*_m_ is the time-averaged ratio between total nanoparticle monomers *N*_m_ and total nanoparticles in a system, $\left\langle F_{m}\right\rangle =\left\langle \frac{N_{m}}{N_{s}}\right\rangle$ . Solvent-accessible surface area (SASA) was computed using VMD^56^ and custom Tcl scripts with a probe radius of 2.64 Å. Lipid-accessible surface area (LASA) and water-accessible surface area (WASA) were calculated using the same spherical probe algorithm by selectively excluding the nonrelevant solvent phase (polar or hydrophobic) and the nanoparticle ligands during selection. Because the SASA probe treats each coarse-grained lipid or water bead independently, the calculated surface areas reflect direct bead–surface contacts, rendering the accessibility measurement independent of overall molecular topology.

#### PMF Analysis

Umbrella sampling simulations were run with the same parameters as the unbiased systems, except that the 2 NPs systems were in a 25 × 25 × 15 nm^3^ box. A steered MD simulation on the NP center of mass was run to generate the initial coordinates. The pull rate was 0.001 nm ps^–1^ and the pull force constant was 750 kJ mol^–1^ nm^-2^. The system was split into 74 windows between a 2.5 and 10 nm center of mass distance between NPs, corresponding to 1 Å wide windows. Each window was simulated for 2 μs, and the center of mass between NPs was restrained by setting the pull rate to 0 nm ps^–1^ while maintaining the pull force constant at 750 kJ mol^–1^ nm^-2^. PMFs were generated using the weighted histogram analysis method (WHAM) implemented in *gromacs’* gmx_wham tool.

## Results and Discussion

### Nanoparticle and Solvent Interactions Are Core Dependent

We first tested whether ligands shield the NP core from interaction with the surrounding environment by simulating a single lipid-embedded, hydrophobically coated NP, varying the NP core bead type. Qualitatively, we observe that NP models with hydrophilic core beads are less embedded in the hydrophobic core of the membrane than their hydrophobic counterparts (Figure 3A,B). To quantify the degree of membrane solvation, we measured the water accessible surface area—the geometric solvent accessible surface area excluding nonwater molecules—of the NP core as a function of ligand length (Figure 3C) or core bead type (Figure 3D). As expected, the water accessible surface area decreases with increasing ligand length due to the increased hydrophobic surface around the NP; the intensity of this effect increases with the hydrophilicity of the core. As shown in Figure 3D, the water accessible surface area increases with the hydrophilicity of the core, irrespective of ligand length, suggesting that the core influences NP–solvent interactions regardless of the ligand coating. This behavior is inconsistent with the common assumption that full surface passivation shields the gold core from interactions with its environment.

**Figure 3:**
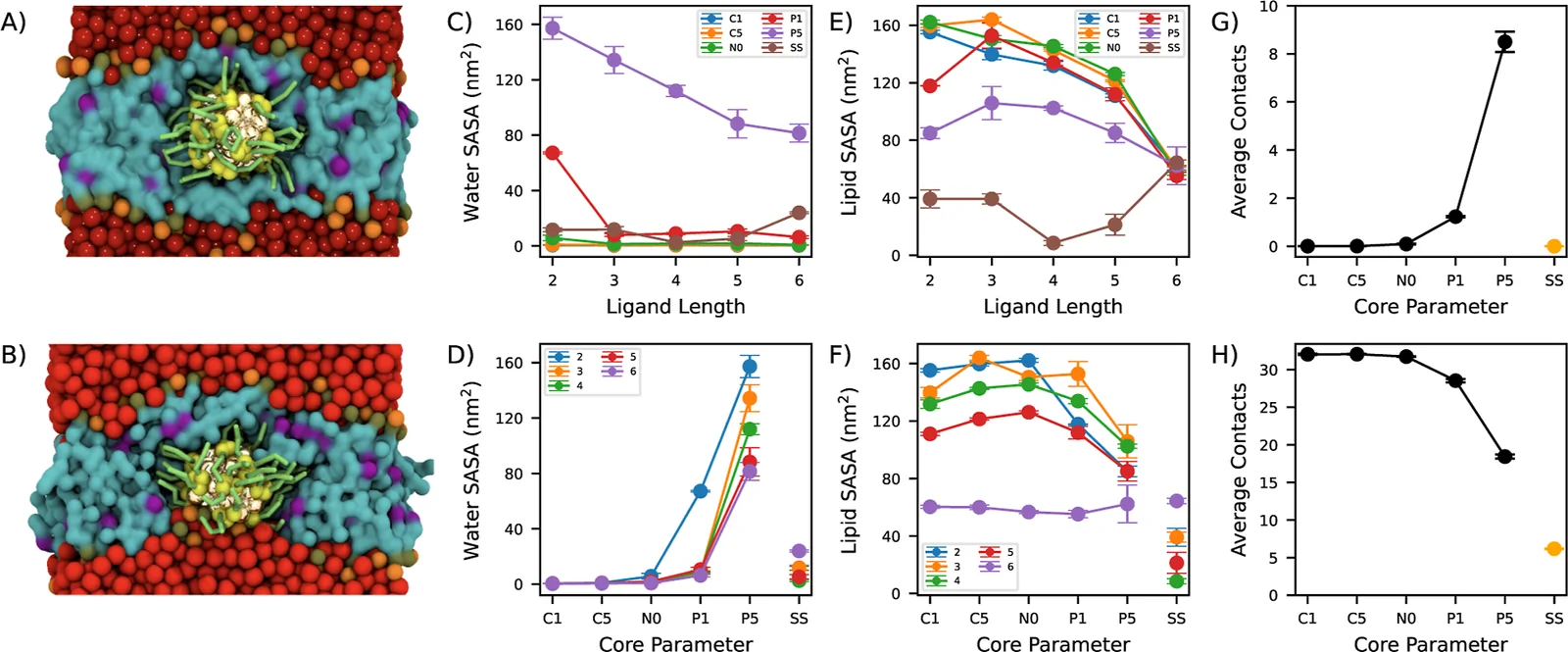
Solvent accessible surface area of NPs with varying compositions. Representative frames of a single nanoparticle composed of hydrophobic C5 (A) or hydrophilic P5 (B) beads embedded in a lipid bilayer. Lipid heads are represented in orange, tails in blue and purple, water in red, nanoparticle core in gold, and ligands in green. Influence of ligand length (C) or core parameter (D) on SASA with respect to the water as solvent. Influence of ligand length (E) or core parameter (F) on SASA with respect to the lipids. Average number of lipid headgroup beads (G) or tailgroup beads (H) with respect to the core parameter. Core parameters are arranged from most to least hydrophobic in Martini (C1, C5, N0, P1, and P5). The SS bead is a non-Martini bead with purely repulsive interactions. Error bars are the standard error of 3 replicas.

To understand the effect of the choice of NP core bead type on lipid–core interactions, we measured the surface area of the NP core accessible to lipids as a function of ligand length (Figure 3E) or core bead type (Figure 3F). NPs are composed of a core and an outer ligand shell; thus, the lipid accessible area is an exposed region on the NP core that is not interacting with the aqueous solvent and is not covered by ligands. Similar to the water accessible surface area, the lipid accessible surface area decreases with increasing ligand length (Figure 3E), except for soft sphere cores. As shown in Figure 3F, the lipid accessible surface area decreases as the core hydrophilicity increases at all ligand lengths except for the longest (tetracosanethiol). The displacement of surrounding lipids is consistent with the increased NP–water interaction shown in Figure 3D. Bilayer-embedded pNPs induce localized structural defects at the water–hydrophobic lipid surface interface, as observed in Figure 3B. Although the membrane can tolerate some defects, high concentrations of hydrophobic AuNPs can hinder vesicle formation by disrupting lipid membranes.^59^

To determine how well models reproduce previously observed lipid–core interactions, we quantify the difference in contact between the heads (Figure 3G) and tails (Figure 3H) of the lipids near the NP core. As shown in Figure 3H, the average lipid tail contacts decrease with increasingly hydrophilic core bead types. Additionally, as the NP core beads become increasingly hydrophilic, the lipid head groups come into contact with the core surface more frequently. This is consistent with an orientation where the lipid headgroup points toward the NP core, as shown in Figure 3B, where we observe the formation of a pore. In the case of soft-sphere cores, both lipid tails and head groups have minimal contact with the NP surface due to purely repulsive interactions. The results for pNPs are consistent with previous atomistic simulations of bare AuNPs at membrane–water interfaces,^32^ which revealed frequent contacts between gold and PC headgroups; we expect lipid headgroups to interact with the core surface, which matches our observations of NP models with more hydrophilic bead types.

### NP Core Parameters Cause Differential Dimerization Pathways

To test whether core parameters affect membrane-mediated interactions between NPs, we measured the free energy of dimerization between NPs consisting of C5 (hNP) or P5 (pNP) core bead types (Figure 4A,B). As shown in Figure 4A, dimerization is stable in both core types, with a free energy of dimerization of −102 ± 3 kJ mol^–1^ for hNP and −97 ± 3 kJ mol^–1^ for pNP. However, the shape of the potential of mean force is distinct, suggesting differing interactions stabilize the hNP and pNP dimers. For instance, the hNP landscape is smooth with a barrier of 12 kJ mol^–1^ when the nanoparticles are separated by 5.5 nm. This is consistent with classic membrane-mediated dimerization of hydrophobic inclusions.^60–62^ In contrast, the pNP landscape lacks any barrier but displays a structured, two stage well and intermediate plateau when nanoparticles are separated by 3.5–4 nm and a narrow deeper well at 3 nm separation. The plateau in free energy may be due to the reconfiguration of the membrane, associated with the closure of pores when the nanoparticles are within a 3 nm distance (Figure 4D); in contrast the free energy of the hNP systems has a single smooth well (Figure 4A). While dimerization of pNPs appears to lack a barrier as a function of the three-dimensional distance *r*, this collective variable may obscure a barrier in the distance projected onto the plane of the membrane: pNPs introduce a pronounced vertical separation at intermediate values of *r* (Figure 4C). This conformation visibly bends the membrane (Figure 4D), but pores opened by the pNPs may offset the bending cost. These pores are unlikely to be favorable for the hNPs because they require interactions between the core and water.

**Figure 4:**
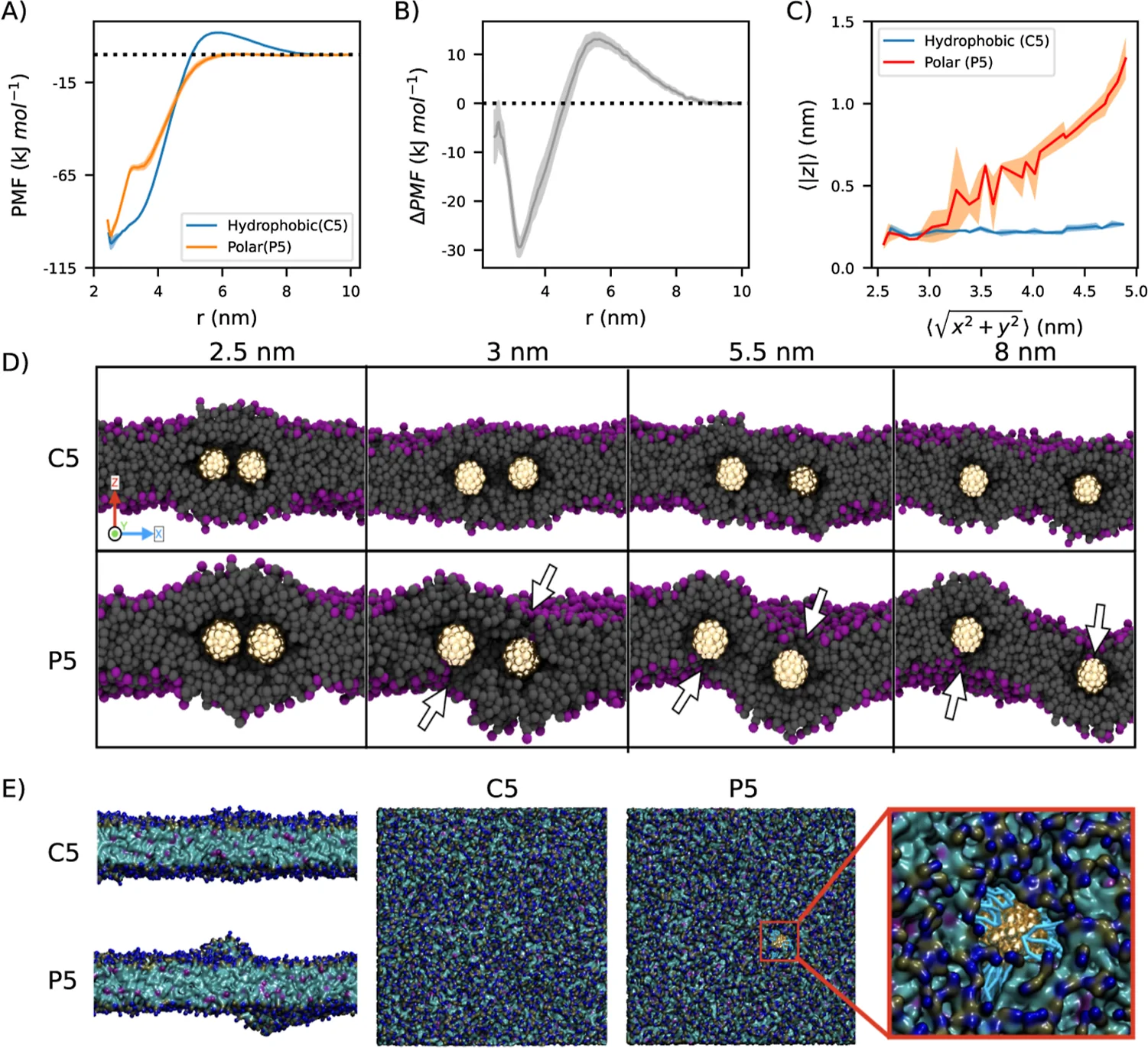
PMF of NP dimerization in NPs with varying core parameters. (A) Dimerization PMFs for NPs comprised of P5 (orange) or C5 (blue) cores. PMFs were calculated as a function of the center of mass distance between two NPs of the same core type. (B) The difference in the dimerization profiles between nanoparticles with P5 and C5 cores. (C) NPs’ two-dimensional *x*–*y* Euclidean distance as a function of the absolute value of the *z* component (axis shown in D). The standard error is plotted in light orange (P5) or blue (C5) along the profile and was calculated using 3 replicas. (D) Representative images of NPs at varying distances. Ligands are not shown. (E) Representative images of the lipid membrane when NPs are at 5.5 nm. Arrows point to membrane pore regions.

### Core Interactions Drive Multinanoparticle Aggregation with Minor Effects from Ligands

To test if NP core parameters affect large-scale nanoparticle aggregation, we simulated systems containing 10 nanoparticles with different core bead types and determined the aggregation propensity (Figure 5). Figure 5A shows representative images of NP aggregation: pNPs readily aggregate, while hNP and NPs with cores comprised of soft sphere (sNP) do not. To quantify the aggregation propensity of NPs and determine the effects of ligand length on nanoparticle aggregation, we measure the nanoparticle fraction in the largest aggregate and monomer fraction (Figure 5B,C). Most pNPs have greater than 50% of NPs in the largest aggregate and fewer than 10% of NPs in monomers (Figure 5B,C). As ligand length increases, the largest aggregate fraction of pNPs approaches that of hNPs and sNPs, becoming indistinguishable at the longest ligand length. This result indicates shielding of the nanoparticle core. In contrast, the monomer fraction for hydrophobic and soft spheres is nonmonotonic, peaking at 4 and 3 bead ligand lengths, respectively (Figure 5C). The fraction of monomers ranges from 40% and 100% in hNP and sNP systems, depending on the ligand length, while the fraction of pNP monomers is unaffected by ligand length. For pNPs, minimization of membrane defects, as shown in Figures 3B and 4C, likely drives pNP aggregation as shown in Figure 5A. In contrast, the monomer fraction of hNPs and sNPs depends nonmonotonically on ligand length, peaking for ligands containing 16–18 and 12–14 hydrocarbon groups, respectively. Because these cores lack the favorable interactions with water associated with membrane defects in pNP systems, aggregation may be more sensitive to subtler collective lipid interactions. Specifically, the monomer-fraction maxima may occur at the ligand lengths for which the NP–ligand geometry best accommodates the leaflet’s preferred local conformation, as determined, for example, by its equilibrium thickness^63^ or monolayer spontaneous curvature.^64^ In such a membrane-mediated mechanism, shorter or longer ligands would impose greater lipid-packing or deformation costs by changing the effective radius of the NP. For hNPs, the membrane-mediated dimerization barrier in Figure 4A is also consistent with this interpretation.

**Figure 5:**
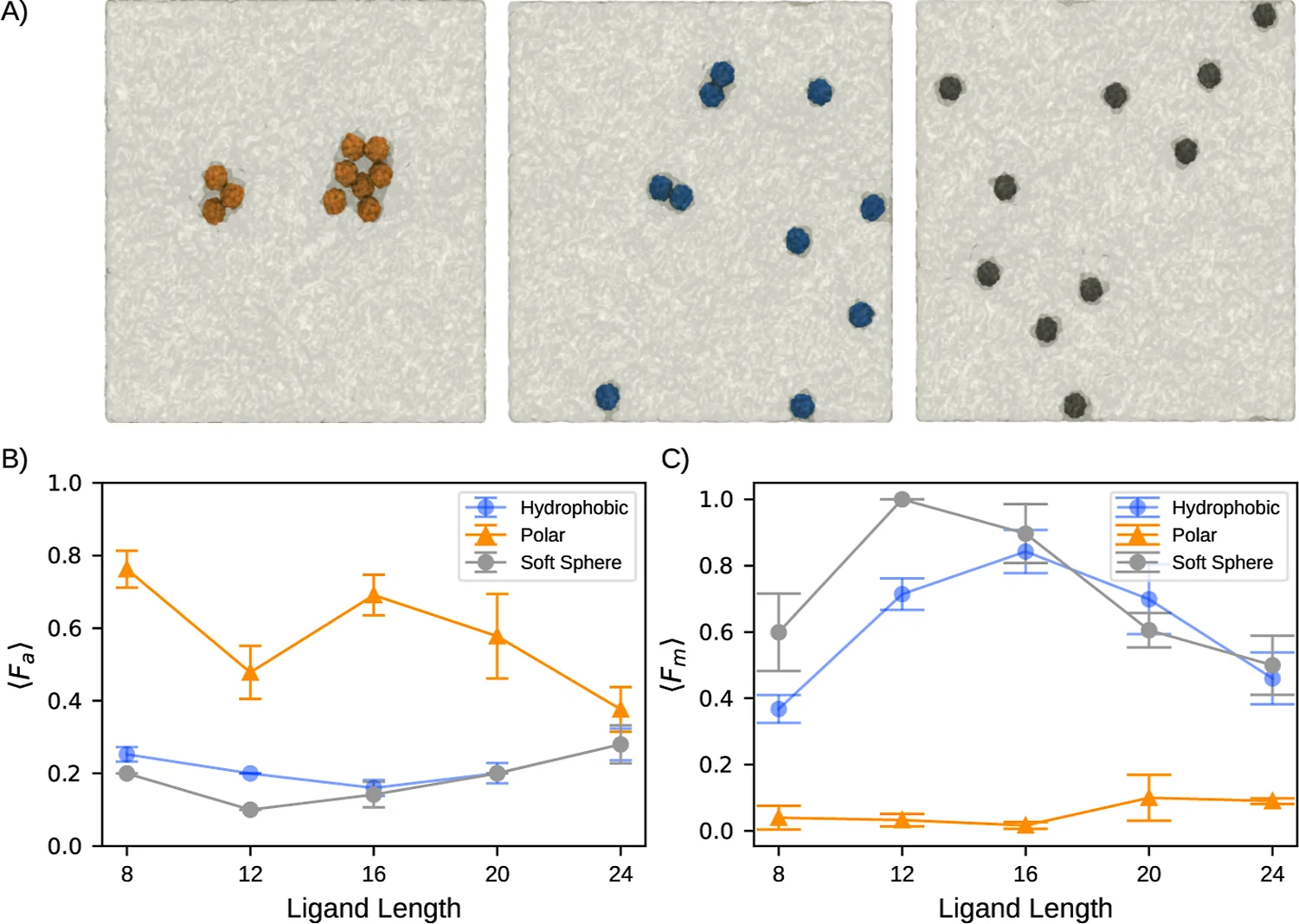
The dependence of aggregate size on NP core parameters and ligand length. (A) Representative images of nanoparticle aggregation of three different core bead types (hydrophilic, hydrophobic, and soft sphere). (B) Fraction of nanoparticles in the largest aggregate and (C) monomer fraction as functions of ligand length. Nanoparticles composed of hydrophilic beads are represented in orange, hydrophobic in blue, and soft spheres in gray. Error bars are the standard error of 5 replicas.

## Conclusion

In this study, we investigated whether the model used for the core of coarse-grained NPs influences NP–NP aggregation within lipid membranes. It is commonly assumed that NP behavior is governed primarily by the outer ligand coating, with the core effectively shielded from interactions with the surrounding environment. However, our results challenge this assumption. Our key findings can be summarized as follows:

1 The mean contact frequency between the NP core and water increases as core hydrophilicity increases. In particular, more hydrophilic cores contact water by exposing the NP core to the aqueous environment through membrane defects.
2 Dimerization kinetics are sensitive to the core model. Dimerization of hNPs exhibits a ∼12 kJ mol^–1^ free-energy barrier corresponding to the transition between the dimerized and undimerized states, consistent with membrane-mediated dimerization.^60–62^ The depth of the free-energy minimum in the fully aggregated state differs between hNP and pNP nanoparticles by approximately 4 ± 5 kJ mol^–1^.
3 Spontaneous large-scale aggregate formation is observed only in the pNP systems, although this behavior is reduced for the longest ligand length (tetracosanethiol). In contrast, hNPs and sNPs predominantly remain as monomers and dimers.

Together, these results suggest that core-dependent solvent interactions may drive aggregation of pNPs, but not hNPs. Because all nanoparticles could induce unfavorable deformations in lipid membranes, NP dimerization—regardless of core type—may reduce the overall free energy of the system. However, in pNPs, exposed core regions interact strongly with the surrounding aqueous solvent, introducing membrane defects. These defects could reduce the energetic barrier for lipid rearrangement and thereby facilitate NP aggregation, ultimately leading to the spontaneous formation of large clusters in pNP systems. In contrast, hNP dimers are unable to induce membrane defects, leading to a barrier to dimerization as the hNPs force the membrane to make a cusp. However, these dimerization results may not be generalizable to many-body interactions because there may be significant differences in the potential of mean force profiles and free energy wells with additional NPs; any such differences are expected to compound with additional NPs.

We observe clear differences in the multiparticle aggregation between the two models, and pNPs, but not hNPs, form multiparticle aggregates, suggesting that core properties are important for understanding experimentally observed aggregation.^13^ However, it is likely that much larger differences would be observed for a coarse-grained gold model that reproduces the strong gold–gold interactions represented in atomistic force fields.^37^ If so, it will be crucial to incorporate such a model into future CG simulations that aim to study gold nanoparticles in membranes.

## Supplementary Material



## Notes

Figure S1: cumulative distributions of aggregate sizes in 10 nanoparticle systems for each ligand length. Figure S2: aggregate size probabilities for systems of 10 or 20 nanoparticles. Figure S3: aggregate size probabilities and cumulative distributions of aggregates for each core type. Figure S4: histograms for umbrella sampling simulations. Figures S5–S9: largest aggregate fraction over time for varying ligand types. Figures S10–S14: monomer fraction over time for varying ligand types. Figures S27–S38: shows the lipid head or tail contact with the nanoparticle surface over time. Figure S39: averaged lipid tail order parameter as a function of distance from the nanoparticle surface. Figure S40 and Tables S1 and S2: ligand and gold nanoparticle parameters (PDF)
