# Supplementary material for: The Core Matters: Aggregation of Neutral Ligand-Coated Nanoparticles in Membranes
Source: J Phys Chem B. 2026 Sep 1;130(37):9496–504. doi: 10.1021/acs.jpcb.6c03349 (PMC13592022; doi:10.1021/acs.jpcb.6c03349)
Supplement: Supplementary file 1 [file jp6c03349_si_001.pdf]

The Core Matters: Aggregation of Neutral Ligand-Coated Nanoparticles in Membranes

Jahmal J. Ennis,[†] Julianne C. Griepenburg,[†,‡] and Grace Brannigan[*,†,‡]

†*Center for Computational and Integrative Biology, Rutgers University-Camden, Camden, New Jersey 08012, USA*

‡*Department of Physics, Rutgers University-Camden, Camden, New Jersey 08012, USA*

E-mail: grace.brannigan@rutgers.edu

Table of Contents

1 PDFs and CDFs **S2**

2 Umbrella sampling histograms **S5**

3 Largest aggregate fraction over time **S6**

4 Monomer fraction over time **S11**

5 Solvent accessible surface areas **S16**

6 Lipid contacts with nanoparticles **S22**

7 Methods **S28**

References **S30**

1 PDFs and CDFs

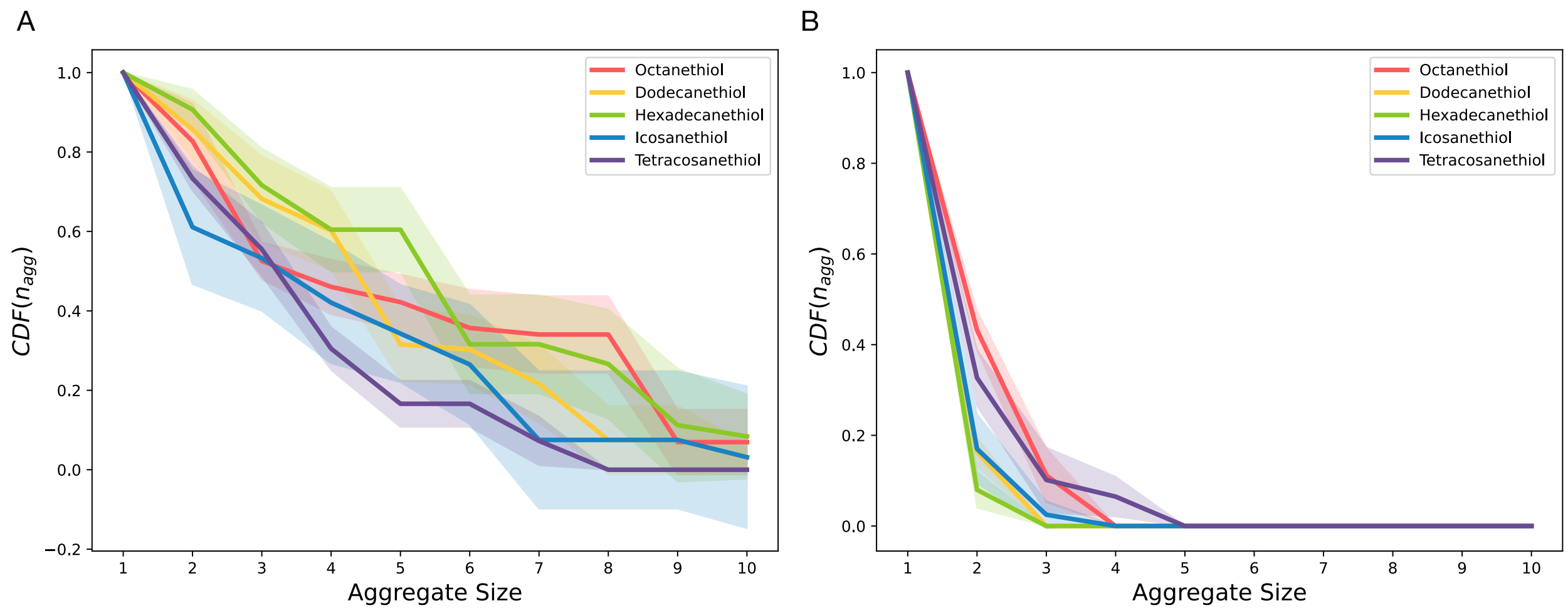

Figure S1: **Comparison of aggregate sizes between nanoparticles of varying ligand length.** Cumulative distribution of (A) hydrophilic nanoparticle (pNP) and (B) hydrophobic nanoparticle (hNP) aggregate sizes. Notably, hNP aggregates do not exceed 5 nanoparticles in size under the conditions tested. The shaded region around each bar represents the standard error of N=5 replicas.

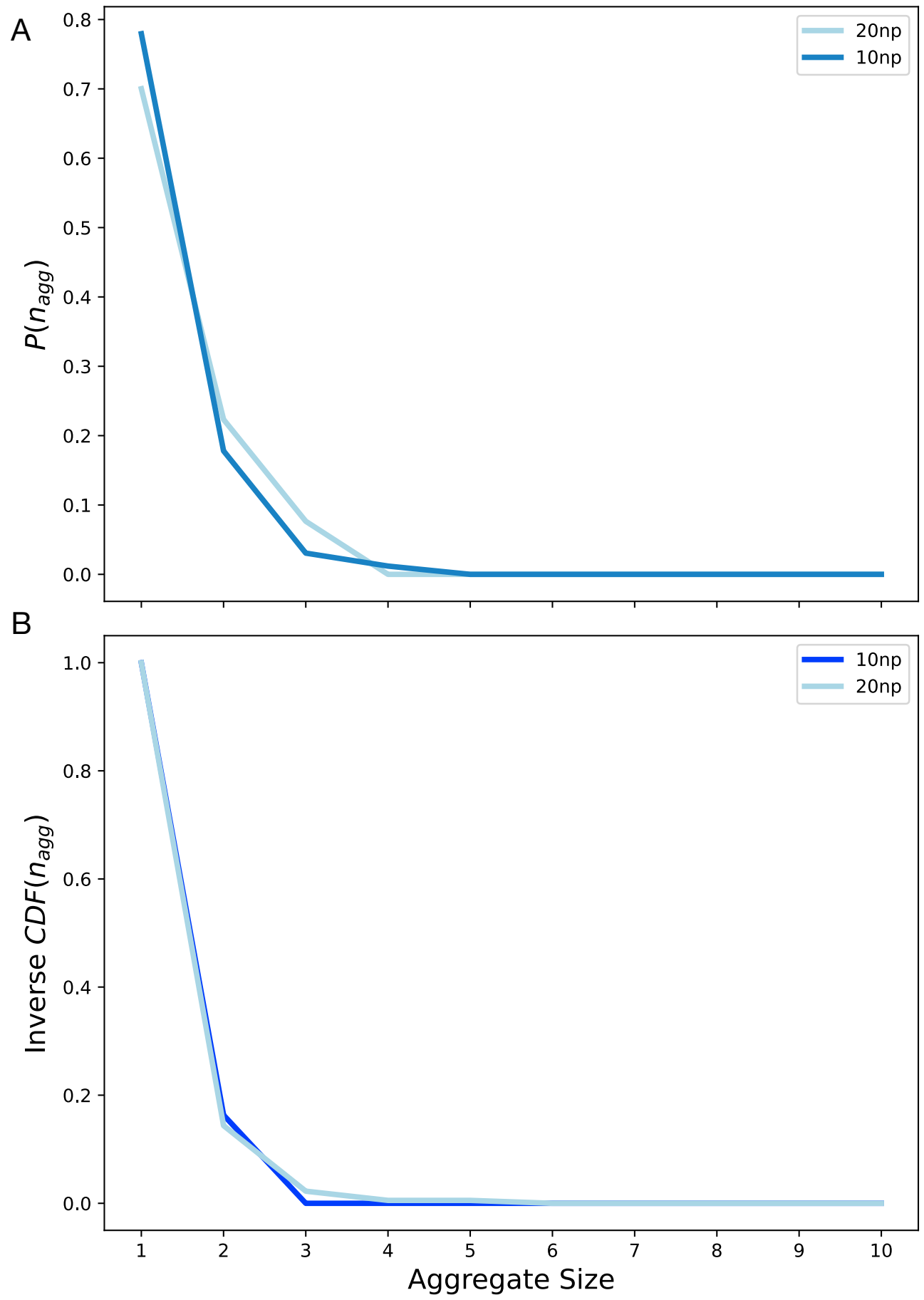

Figure S2: **Validation of nanoparticle concentration effects on aggregation.** (A) Probability distribution and (B) complementary cumulative distribution of aggregate sizes for hydrophobic nanoparticles (hNP). Results are shown for systems containing 10 (dark blue) and 20 (light blue) nanoparticles. Data are pooled across all ligand lengths tested (C8–C24).

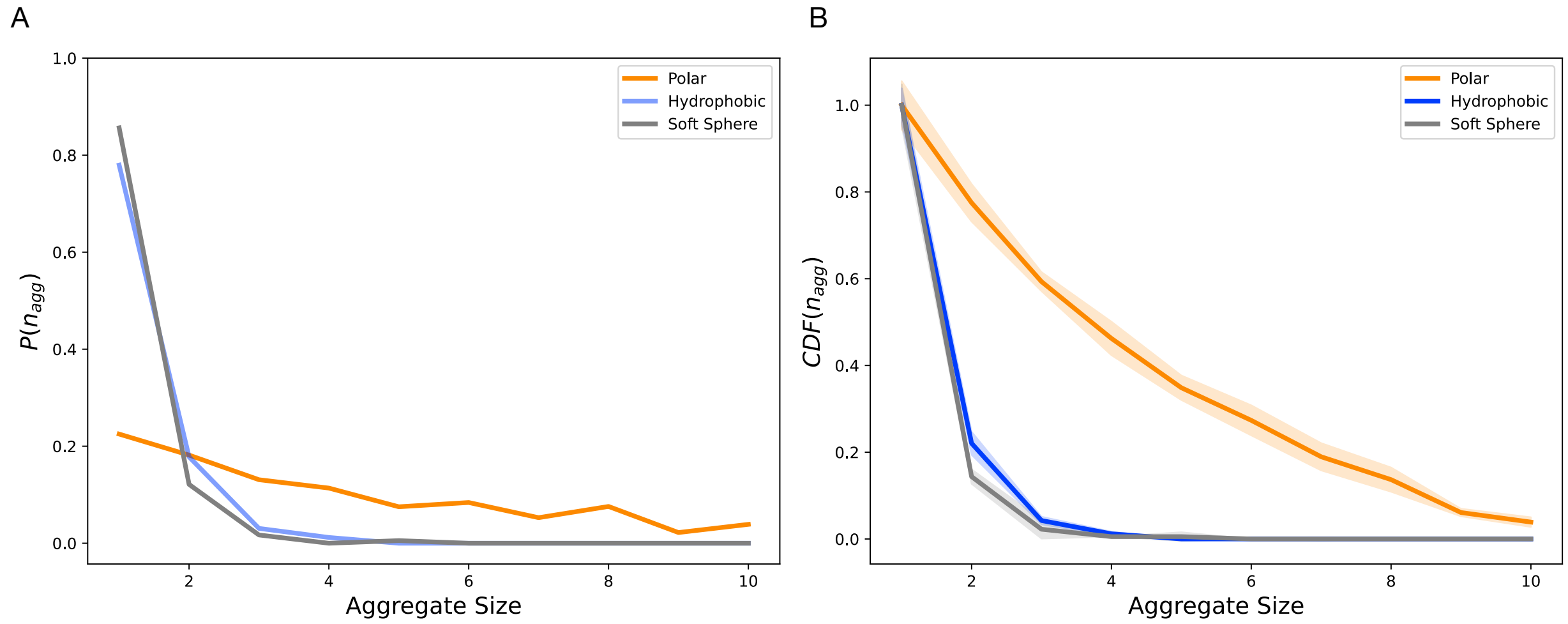

Figure S3: **Impact of core bead-type on nanoparticle aggregation.** (A) Probability distribution and (B) cumulative distribution of aggregate sizes for hydrophilic (pNP, orange), hydrophobic (hNP, blue), and soft-sphere (sNP, grey) core models. Shaded regions represent the standard error across N=5 replicas. Data are pooled across all ligand lengths (C8–C24)

2 Umbrella sampling histograms

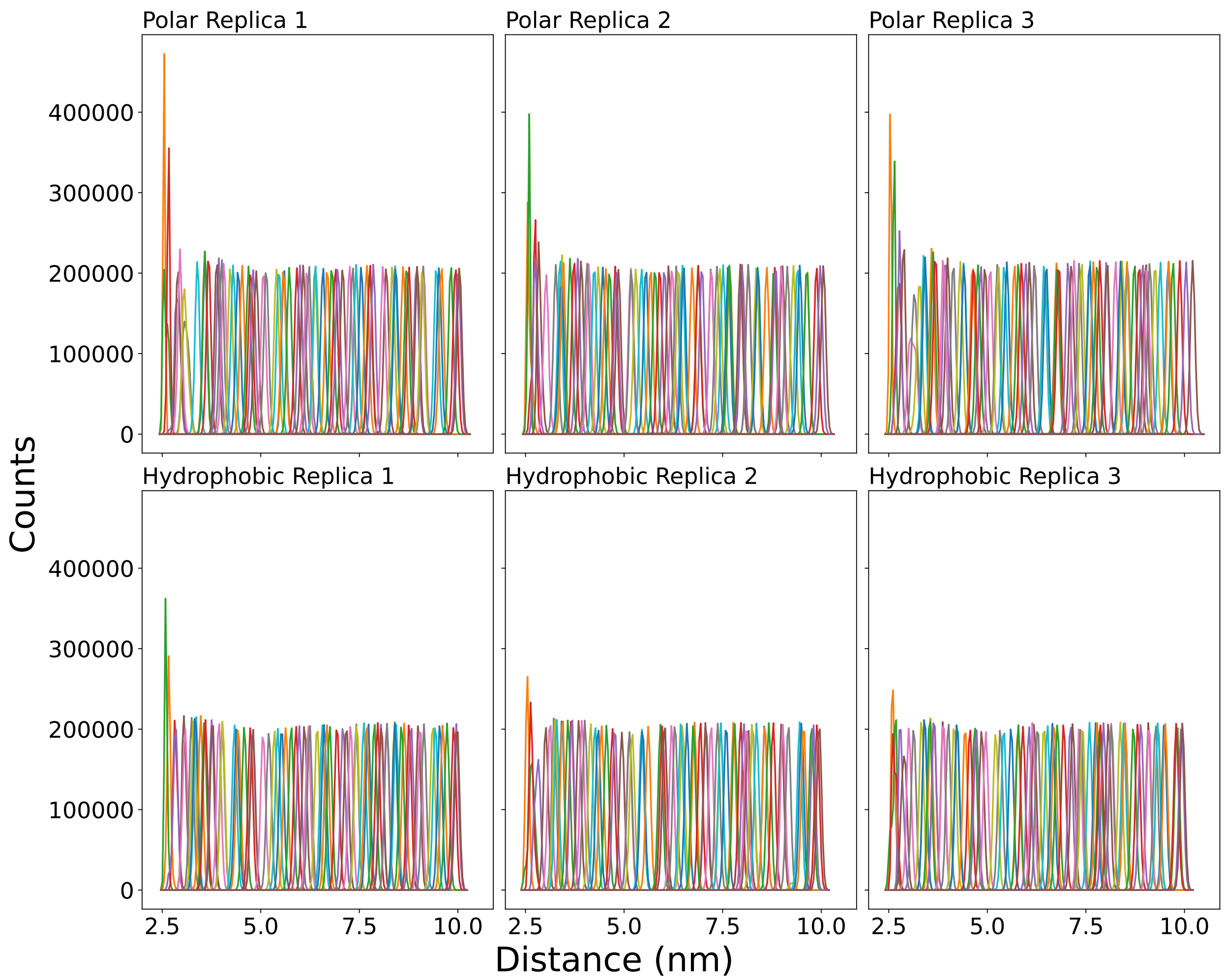

Figure S4: **Biased reaction coordinate sampling for free energy reconstruction.** Histograms show the probability distribution of the center-of-mass distance between two nanoparticles with hydrophilic (top, pNP) and hydrophobic (bottom, hNP) cores. Data were collected from 74 consecutive umbrella sampling windows, each utilizing a harmonic restraining potential with a force constant of $750\,\mathrm{kJ\,mol^{-1}\,nm^{-2}}$. The distributions demonstrate sufficient overlap for free energy reconstruction via the Weighted Histogram Analysis Method (WHAM).

3 Largest aggregate fraction over time

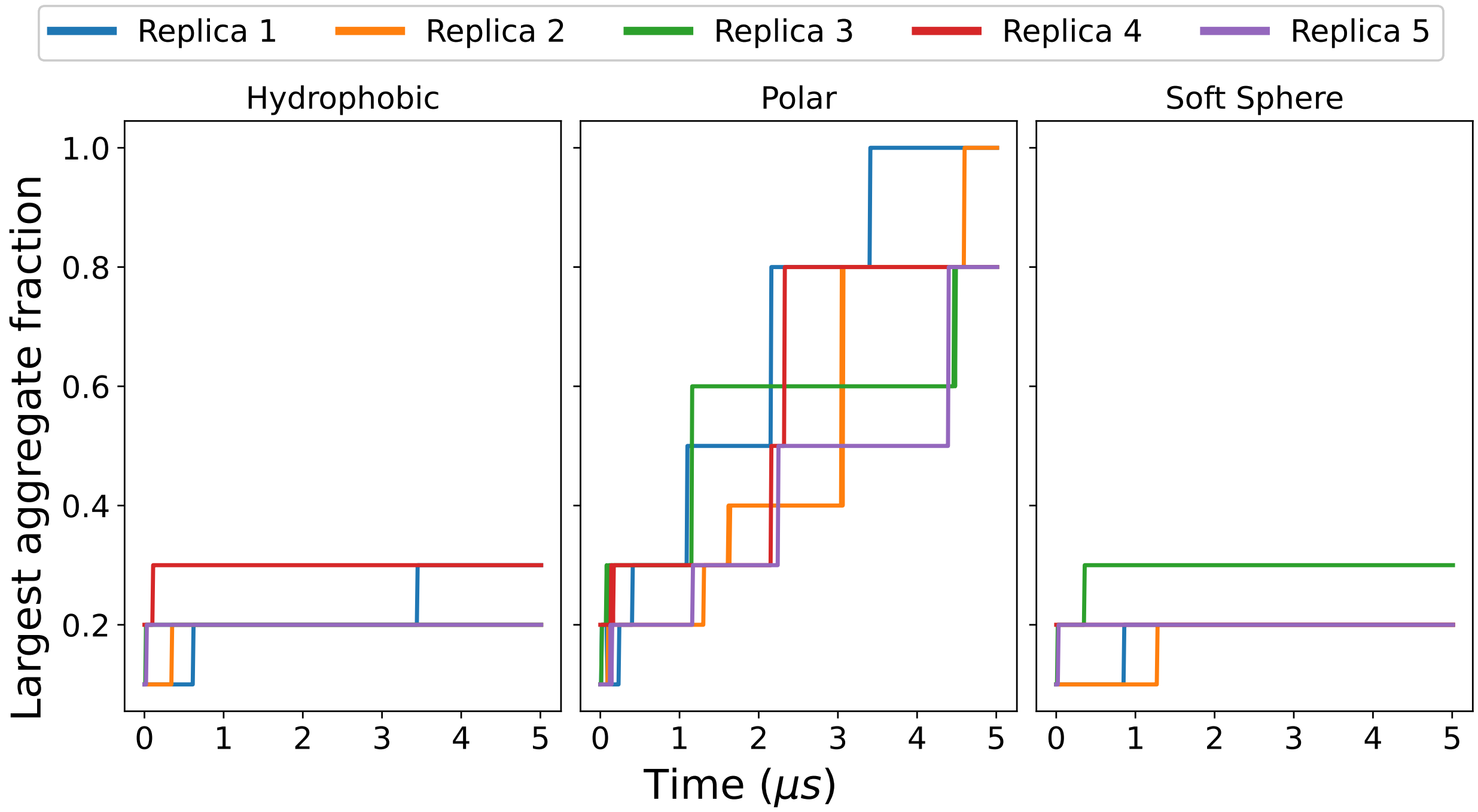

Figure S5: **Convergence of largest aggregate size fraction for C8-ligated (octanethiol) nanoparticles.** Time series of the largest aggregate fraction for hydrophobic (hNP, left), hydrophilic (pNP, middle), and soft-sphere (sNP, right) models over 5 µs. Each panel displays N=5 independent replicas (colored lines). The consistency across replicas indicates that the systems have reached a stationary state regarding the largest aggregate size fraction.

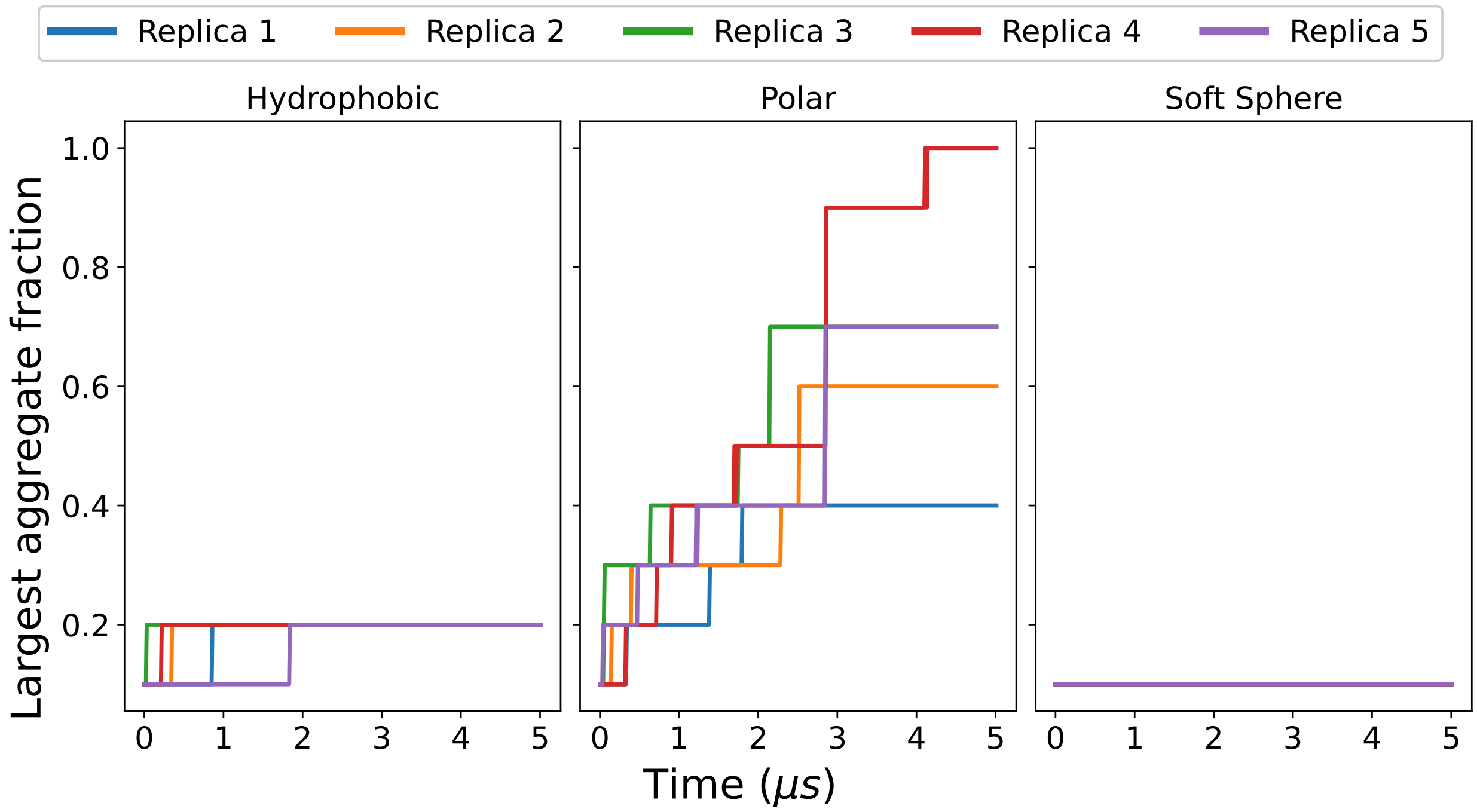

Figure S6: **Convergence of largest aggregate size fraction for C12-ligated (dodecanethiol) nanoparticles.** Time series of the largest aggregate fraction for hydrophobic (hNP, left), hydrophilic (pNP, middle), and soft-sphere (sNP, right) models over 5 µs. Each panel displays N=5 independent replicas (colored lines). The consistency across replicas indicates that the systems have reached a stationary state regarding the largest aggregate size fraction.

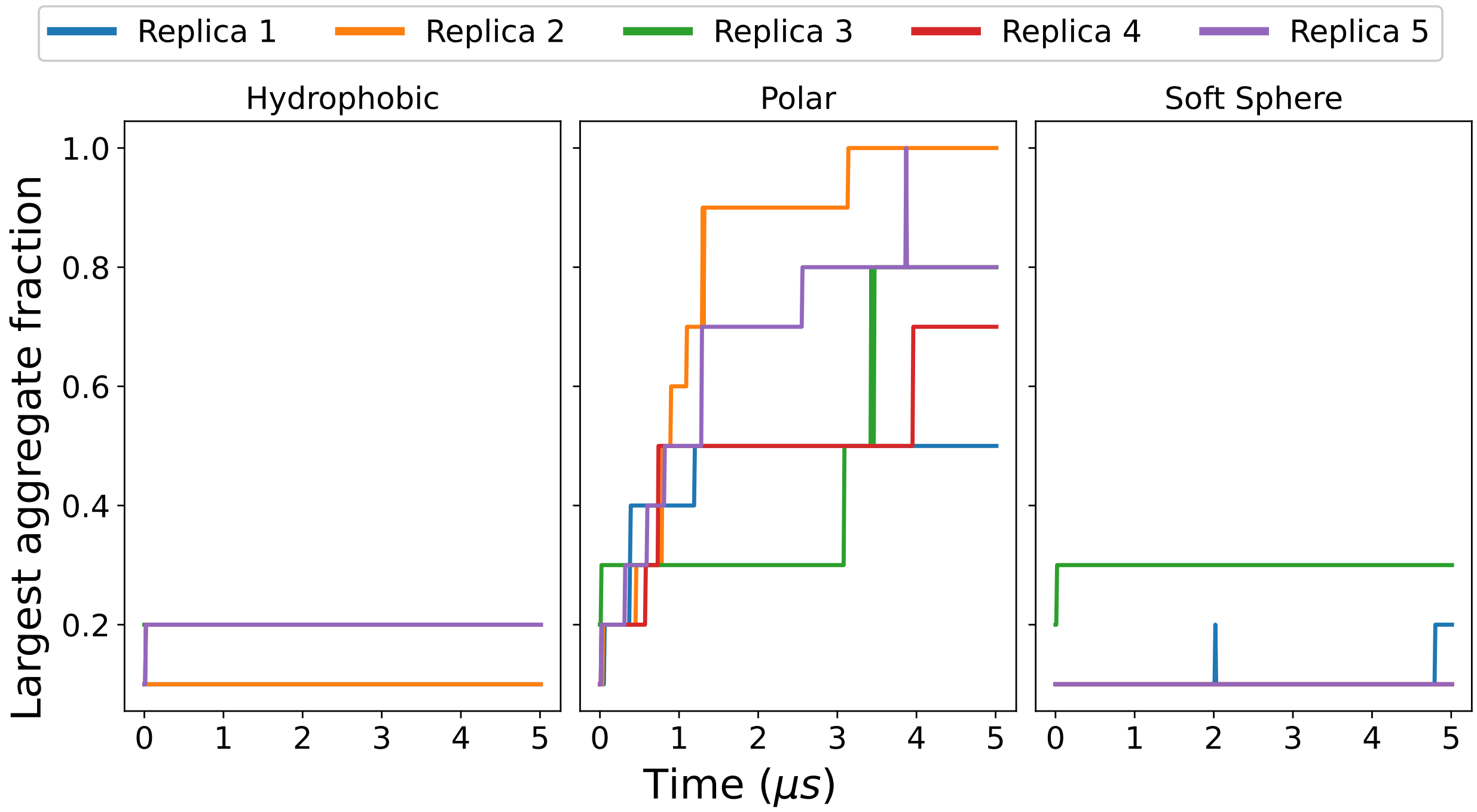

Figure S7: **Convergence of largest aggregate size fraction for C16-ligated (hexadecanethiol) nanoparticles.** Time series of the largest aggregate fraction for hydrophobic (hNP, left), hydrophilic (pNP, middle), and soft-sphere (sNP, right) models over 5 µs. Each panel displays N=5 independent replicas (colored lines). The consistency across replicas indicates that the systems have reached a stationary state regarding the largest aggregate size fraction.

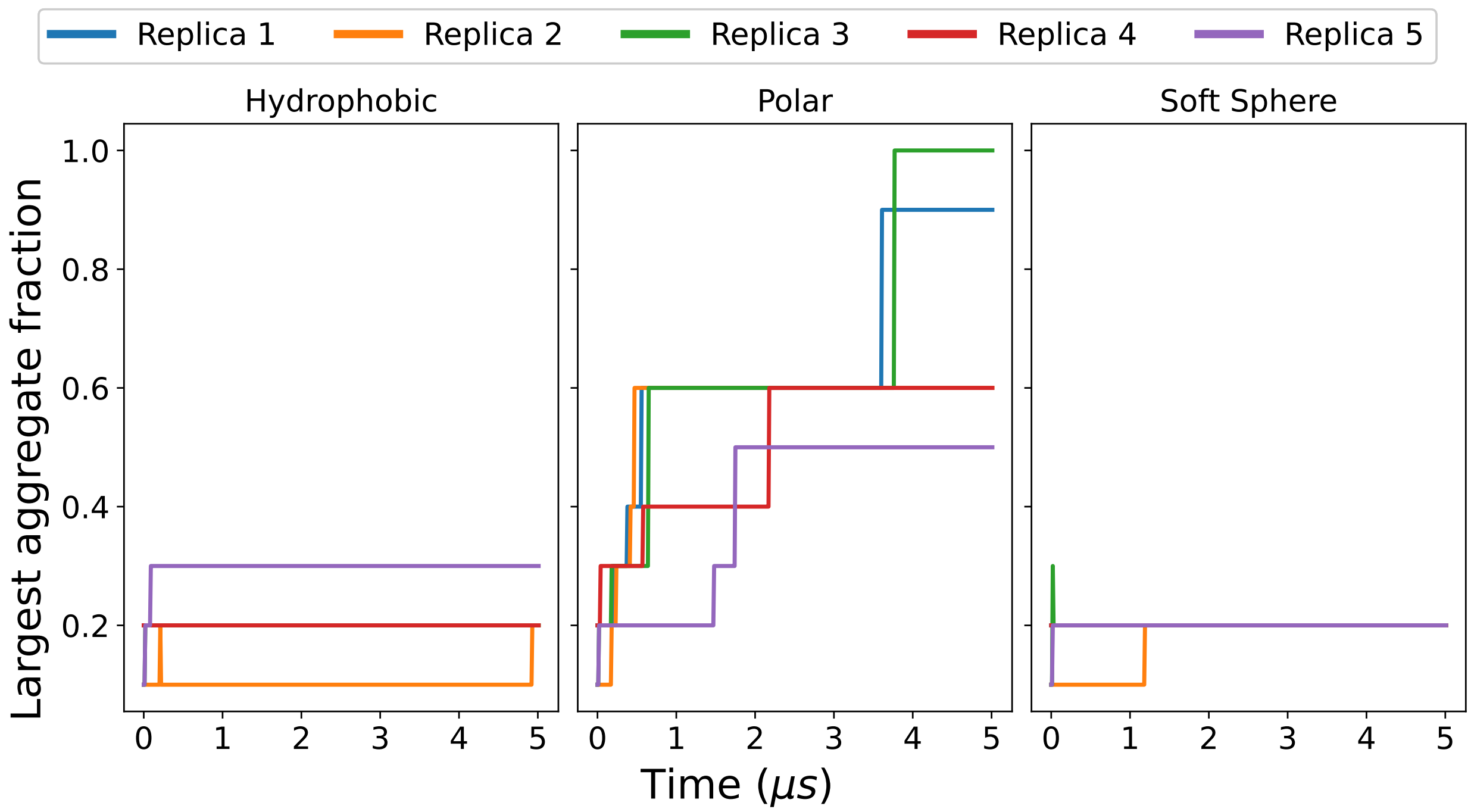

Figure S8: **Convergence of largest aggregate size fraction for C20-ligated (icosanethiol) nanoparticles.** Time series of the largest aggregate fraction for hydrophobic (hNP, left), hydrophilic (pNP, middle), and soft-sphere (sNP, right) models over 5 µs. Each panel displays N=5 independent replicas (colored lines). The consistency across replicas indicates that the systems have reached a stationary state regarding the largest aggregate size fraction.

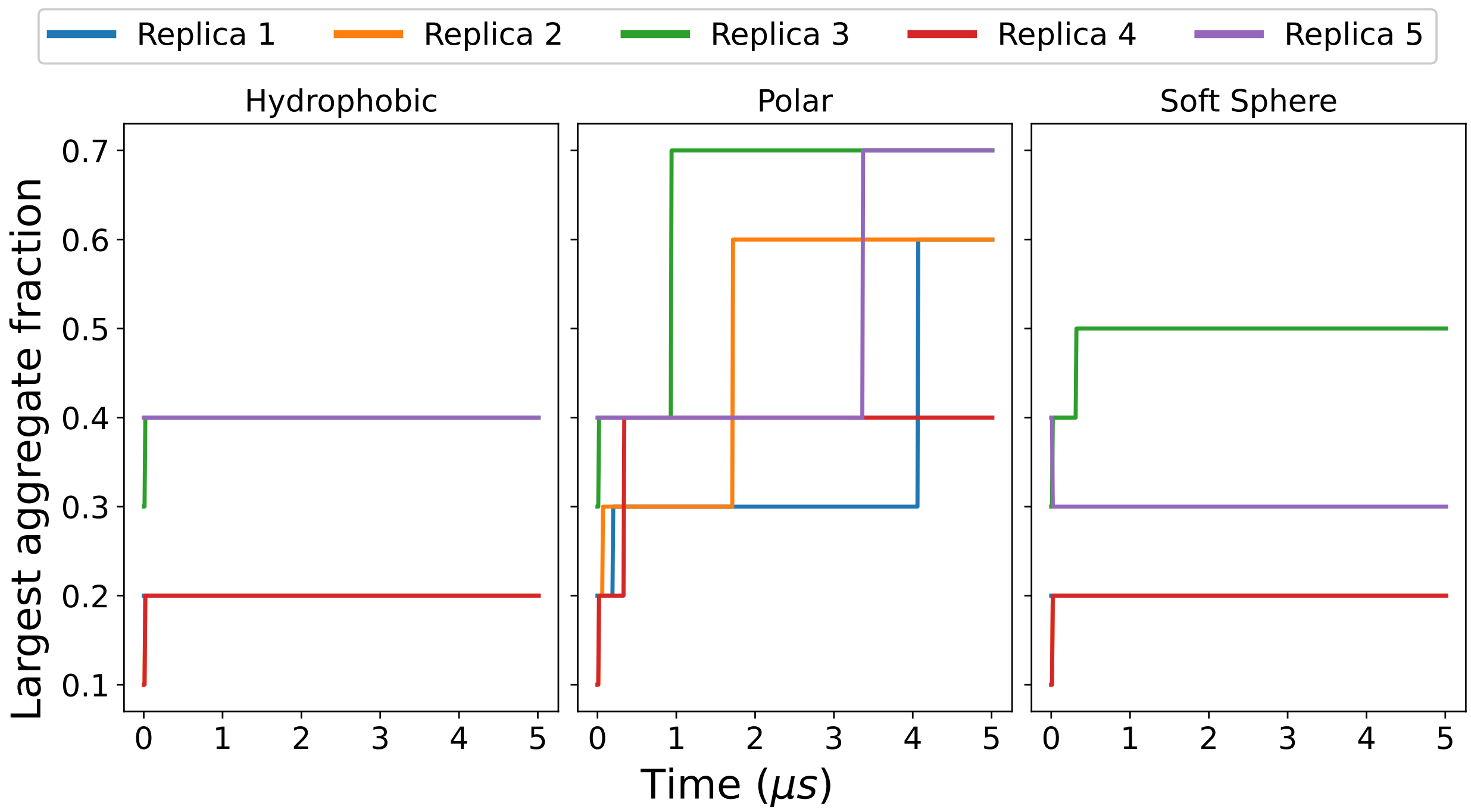

Figure S9: **Convergence of largest aggregate size fraction for C24-ligated (tetracosanethiol) nanoparticles.** Time series of the largest aggregate fraction for hydrophobic (hNP, left), hydrophilic (pNP, middle), and soft-sphere (sNP, right) models over 5 µs. Each panel displays N=5 independent replicas (colored lines). The consistency across replicas indicates that the systems have reached a stationary state regarding largest aggregate size fraction.

4 Monomer fraction over time

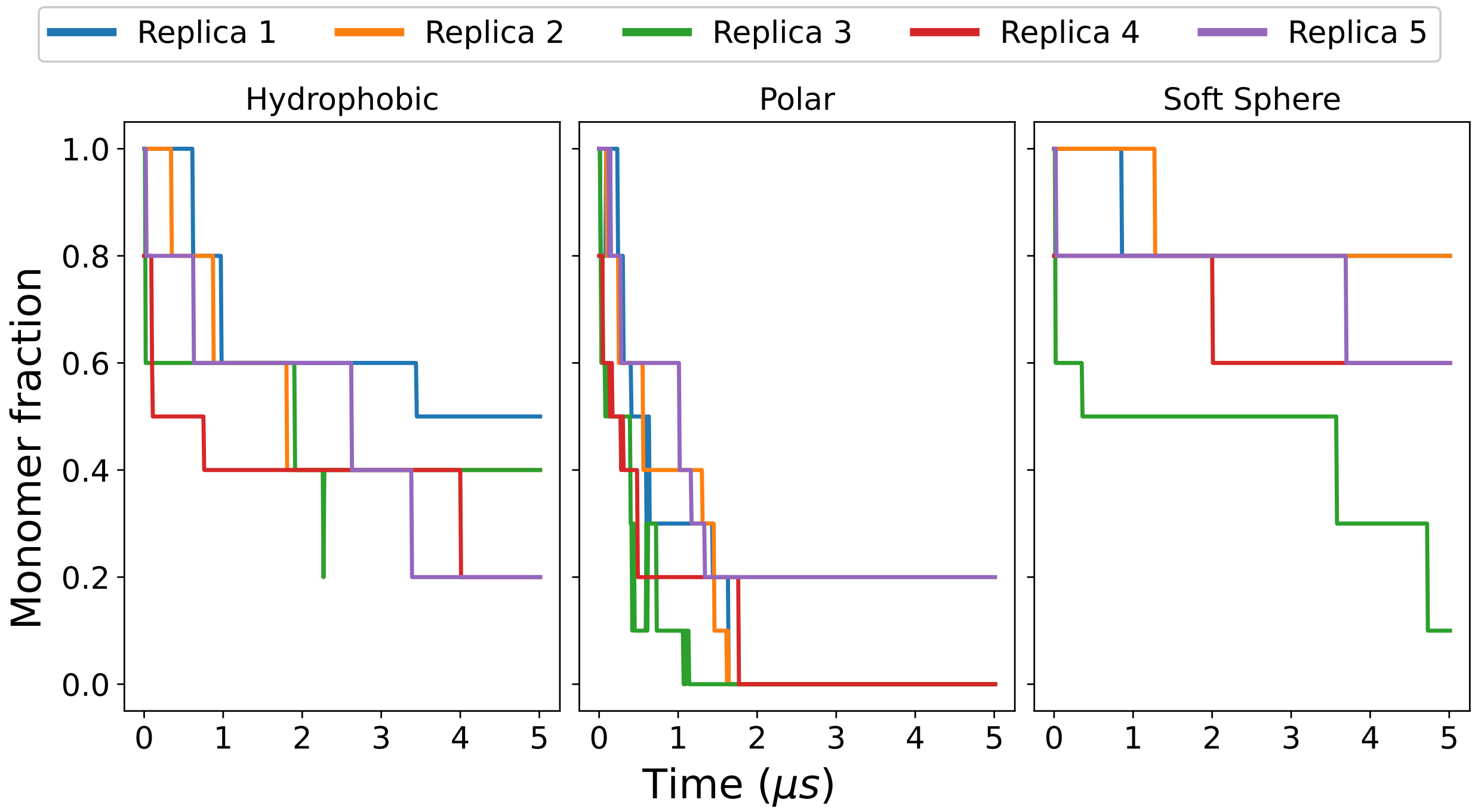

Figure S10: **Convergence of the monomer fraction for C8-ligated (octanethiol) nanoparticles.** Time series of the monomer fraction for hydrophobic (hNP, left), hydrophilic (pNP, middle), and soft-sphere (sNP, right) models over 5 µs. Each panel displays N=5 independent replicas (colored lines). The consistency across replicas indicates that the systems have reached a stationary state regarding the monomer fraction.

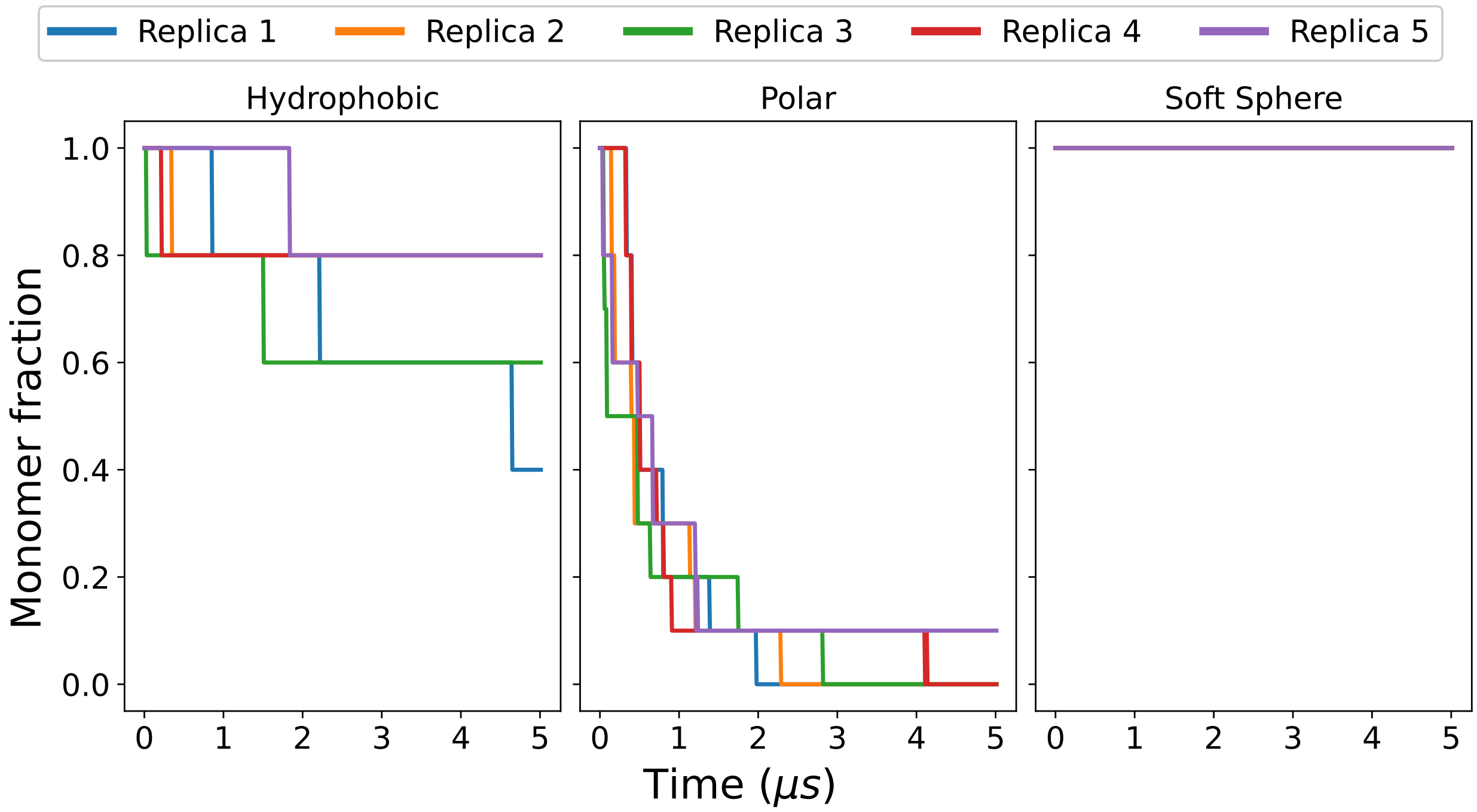

Figure S11: **Convergence of the monomer fraction for C12-ligated (dodecanethiol) nanoparticles.** Time series of the monomer fraction for hydrophobic (hNP, left), hydrophilic (pNP, middle), and soft-sphere (sNP, right) models over 5 μs. Each panel displays N=5 independent replicas (colored lines). The consistency across replicas indicates that the systems have reached a stationary state regarding the monomer fraction.

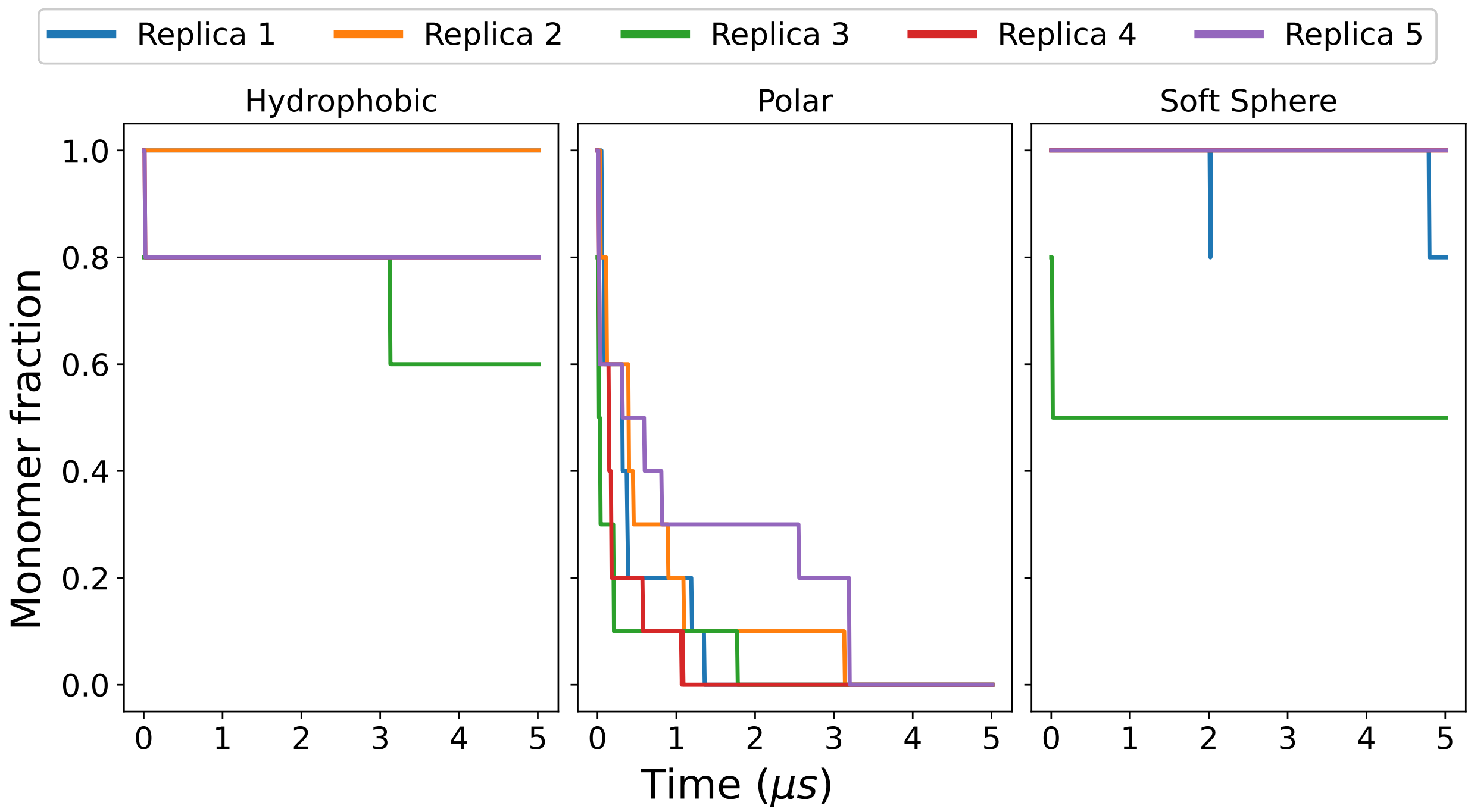

Figure S12: **Convergence of the monomer fraction for C16-ligated (hexadecanethiol) nanoparticles.** Time series of the monomer fraction for hydrophobic (hNP, left), hydrophilic (pNP, middle), and soft-sphere (sNP, right) models over 5 µs. Each panel displays N=5 independent replicas (colored lines). The consistency across replicas indicates that the systems have reached a stationary state regarding the monomer fraction.

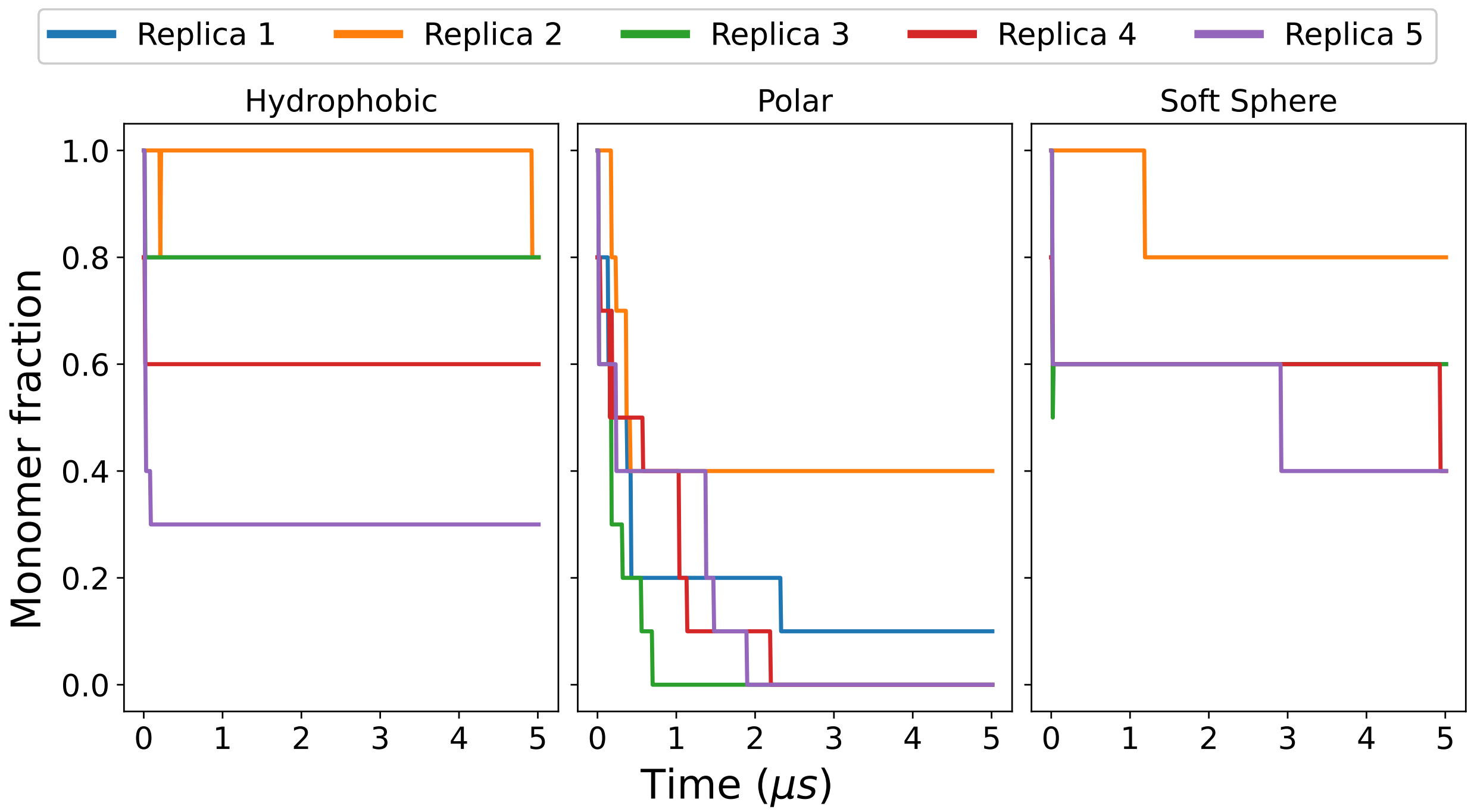

Figure S13: **Convergence of the monomer fraction for C20-ligated (icosanethiol) nanoparticles.** Time series of the monomer fraction for hydrophobic (hNP, left), hydrophilic (pNP, middle), and soft-sphere (sNP, right) models over 5 µs. Each panel displays N=5 independent replicas (colored lines). The consistency across replicas indicates that the systems have reached a stationary state regarding the monomer fraction.

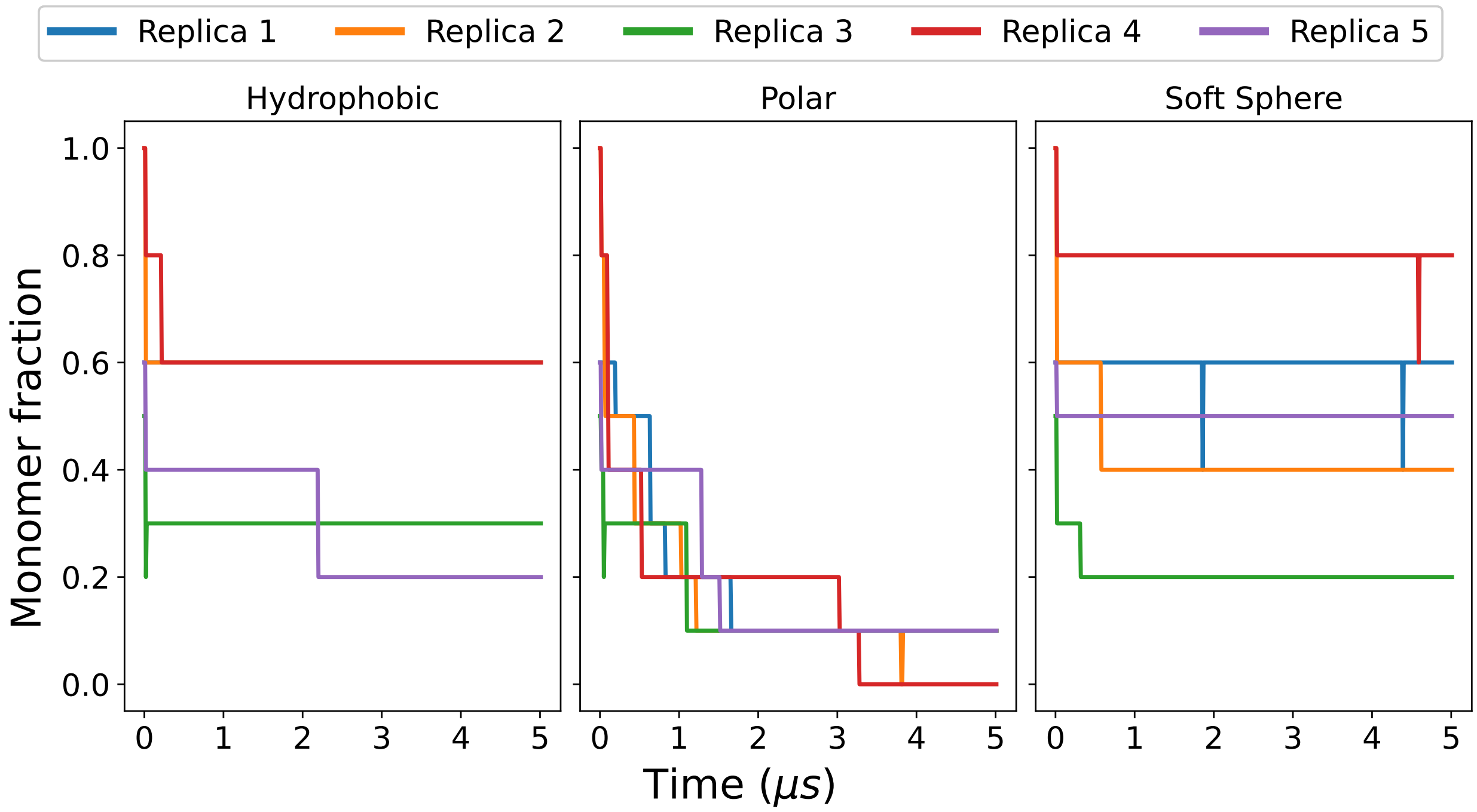

Figure S14: **Convergence of the monomer fraction for C24-ligated (tetracosanethiol) nanoparticles.** Time series of the monomer fraction for hydrophobic (hNP, left), hydrophilic (pNP, middle), and soft-sphere (sNP, right) models over 5 µs. Each panel displays N=5 independent replicas (colored lines). The consistency across replicas indicates that the systems have reached a stationary state regarding the monomer fraction.

5 Solvent accessible surface areas

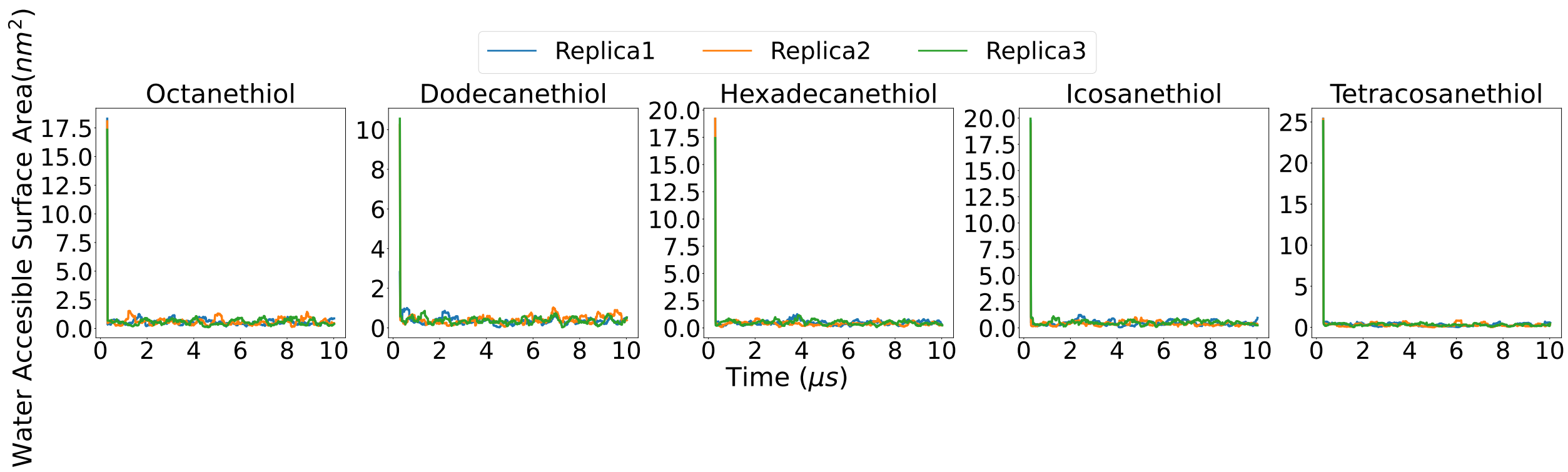

Figure S15: **Convergence of Solvent Accessible Surface Area (SASA) for nanoparticles with C1 bead cores.** Time series of the water-accessible surface area for nanoparticles with ligand lengths ranging from C8 (octanethiol) to C24 (tetracosanthiol). Each panel displays N=3 independent replicas (colored lines); data are presented as a 300-ns moving average. The consistency across replicas indicates that the systems have reached a stationary state with respect to the SASA.

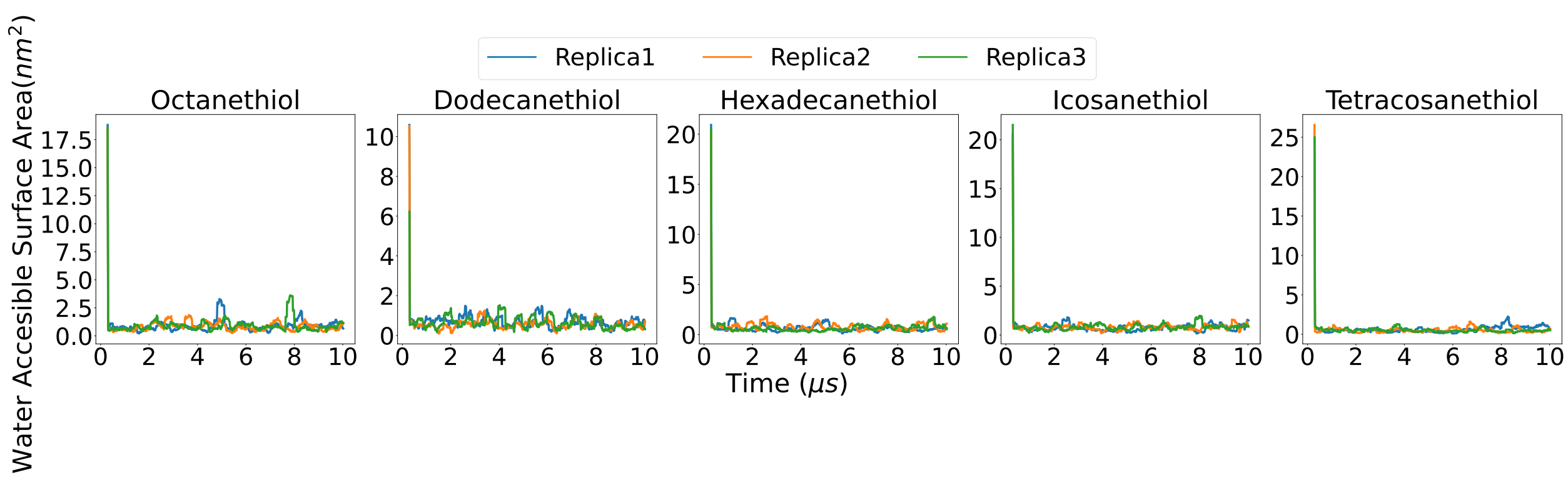

Figure S16: **Convergence of Solvent Accessible Surface Area (SASA) for nanoparticles with C5 bead cores.** Time series of the water-accessible surface area for nanoparticles with ligand lengths ranging from C8 (octanethiol) to C24 (tetracosanthiol). Each panel displays N=3 independent replicas (colored lines); data are presented as a 300-ns moving average. The consistency across replicas indicates that the systems have reached a stationary state with respect to the SASA.

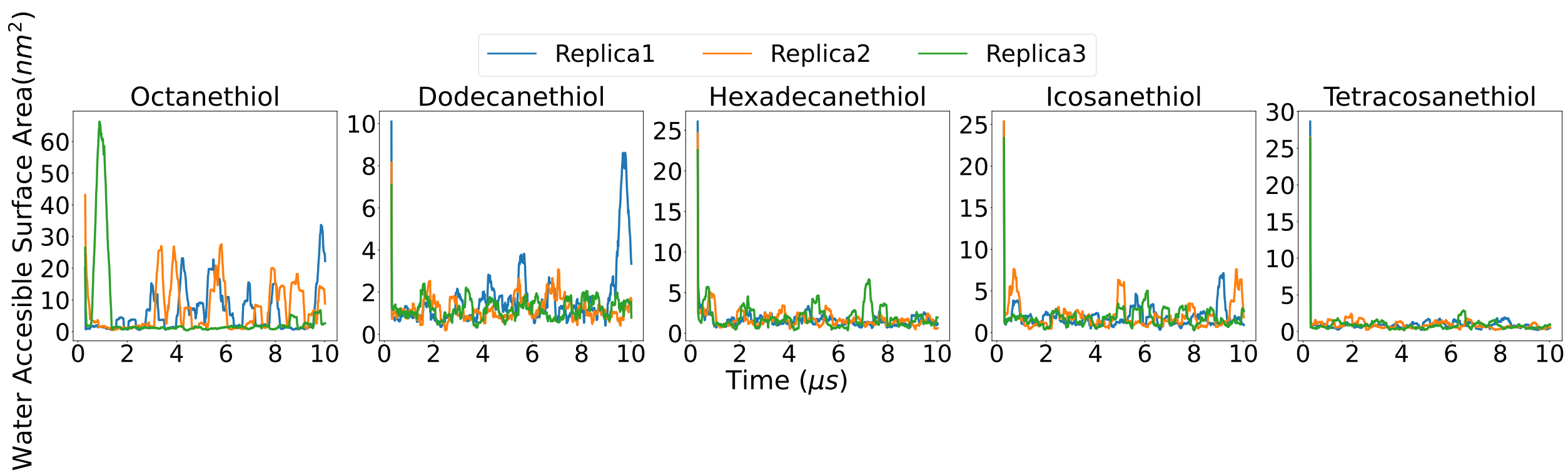

Figure S17: **Convergence of Solvent Accessible Surface Area (SASA) for nanoparticles with N0 bead cores.** Time series of the water-accessible surface area for nanoparticles with ligand lengths ranging from C8 (octanethiol) to C24 (tetracosanthiol). Each panel displays N=3 independent replicas (colored lines); data are presented as a 300-ns moving average. The consistency across replicas indicates that the systems have reached a stationary state with respect to the SASA.

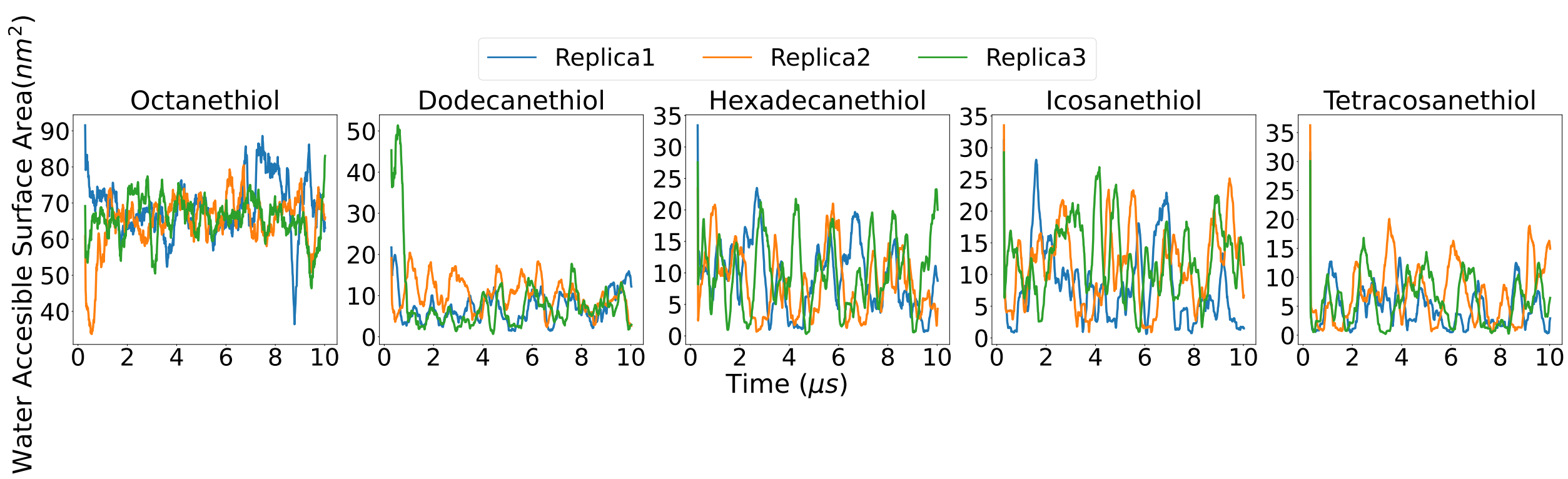

Figure S18: **Convergence of Solvent Accessible Surface Area (SASA) for nanoparticles with P1 bead cores.** Time series of the water-accessible surface area for nanoparticles with ligand lengths ranging from C8 (octanethiol) to C24 (tetracosanthiol). Each panel displays N=3 independent replicas (colored lines); data are presented as a 300-ns moving average. The consistency across replicas indicates that the systems have reached a stationary state with respect to the SASA.

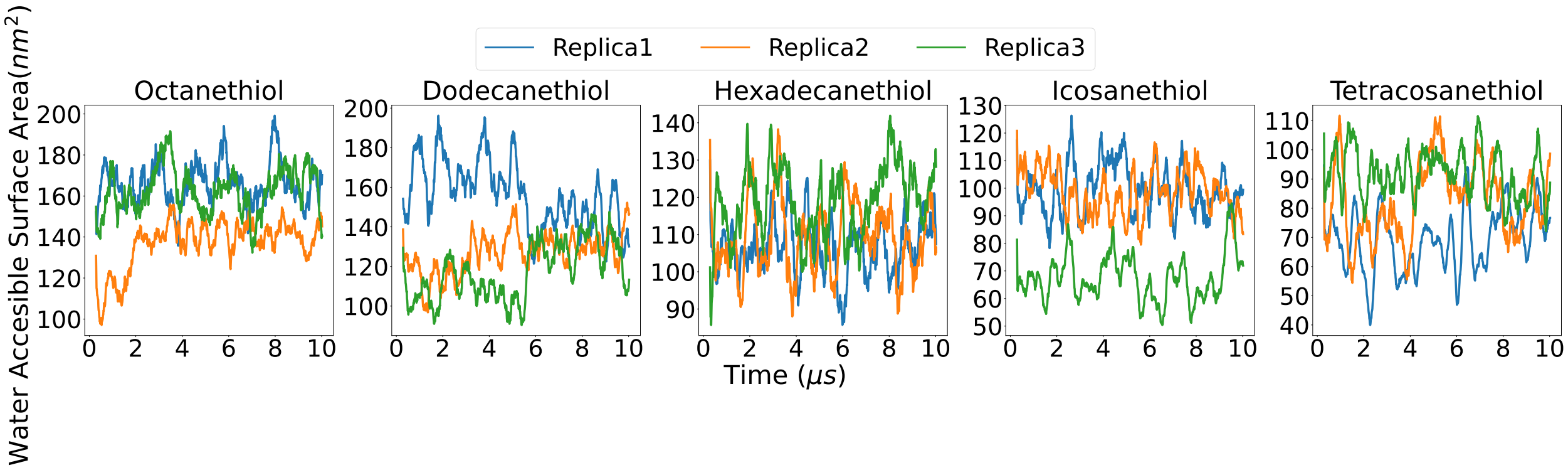

Figure S19: **Convergence of Solvent Accessible Surface Area (SASA) for nanoparticles with P5 bead cores.** Time series of the water-accessible surface area for nanoparticles with ligand lengths ranging from C8 (octanethiol) to C24 (tetracosanthiol). Each panel displays N=3 independent replicas (colored lines); data are presented as a 300-ns moving average. The consistency across replicas indicates that the systems have reached a stationary state with respect to the SASA.

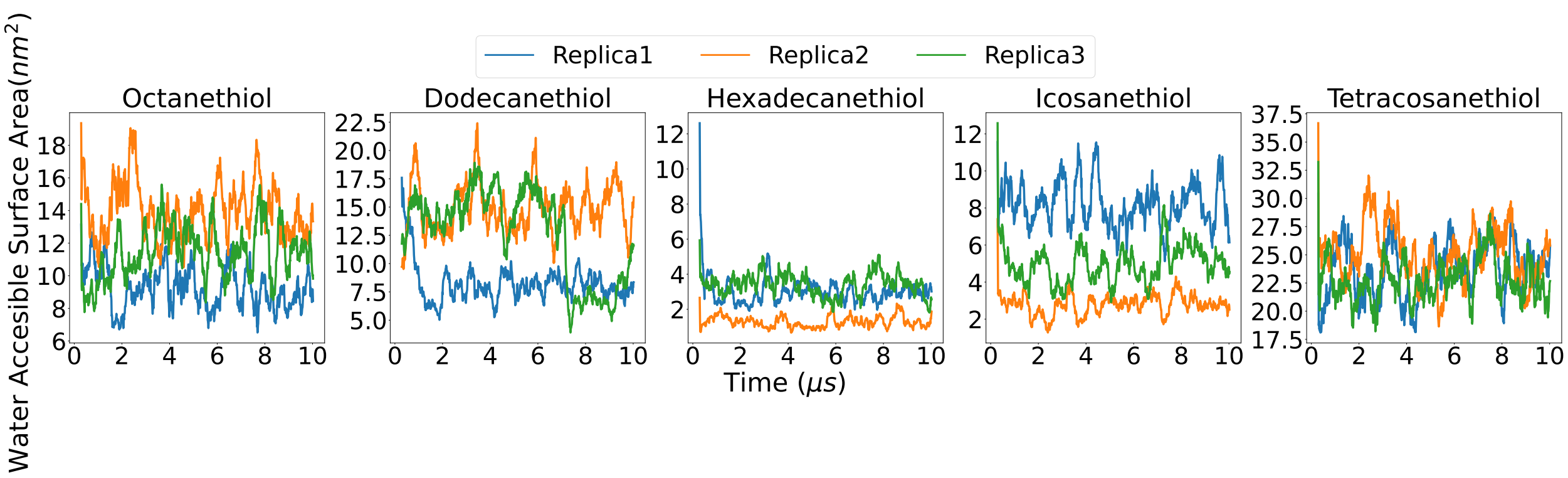

Figure S20: **Convergence of Solvent Accessible Surface Area (SASA) for nanoparticles with SS bead cores.** Time series of the water-accessible surface area for nanoparticles with ligand lengths ranging from C8 (octanethiol) to C24 (tetracosanthiol). Each panel displays N=3 independent replicas (colored lines); data are presented as a 300-ns moving average. The consistency across replicas indicates that the systems have reached a stationary state with respect to the SASA.

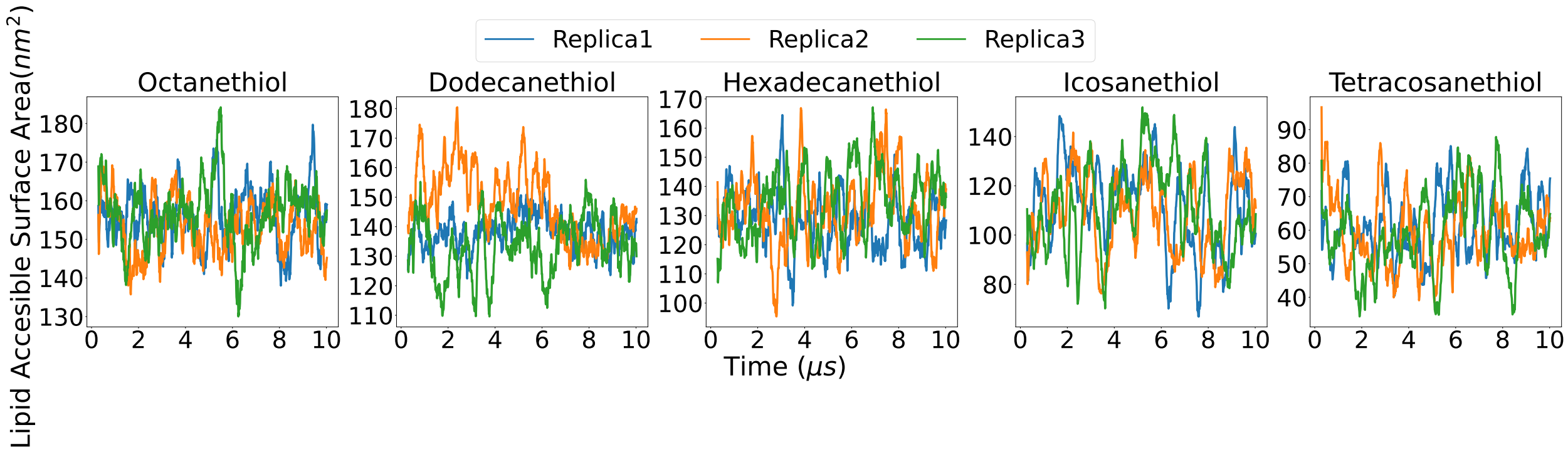

Figure S21: **Convergence of Solvent Accessible Surface Area (SASA) for nanoparticles with C1 bead cores.** Time series of the lipid-accessible surface area for nanoparticles with ligand lengths ranging from C8 (octanethiol) to C24 (tetracosanthiol). Each panel displays N=3 independent replicas (colored lines); data are presented as a 300-ns moving average. The consistency across replicas indicates that the systems have reached a stationary state with respect to the SASA.

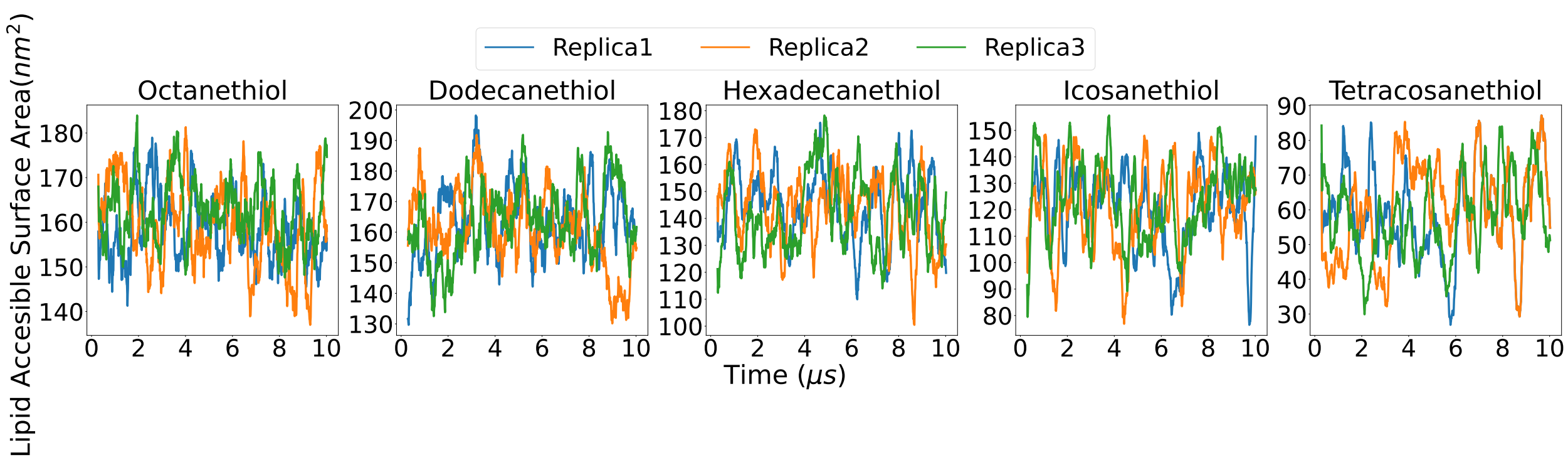

Figure S22: **Convergence of Solvent Accessible Surface Area (SASA) for nanoparticles with C5 bead cores.** Time series of the lipid-accessible surface area for nanoparticles with ligand lengths ranging from C8 (octanethiol) to C24 (tetracosanthiol). Each panel displays N=3 independent replicas (colored lines); data are presented as a 300-ns moving average. The consistency across replicas indicates that the systems have reached a stationary state with respect to the SASA.

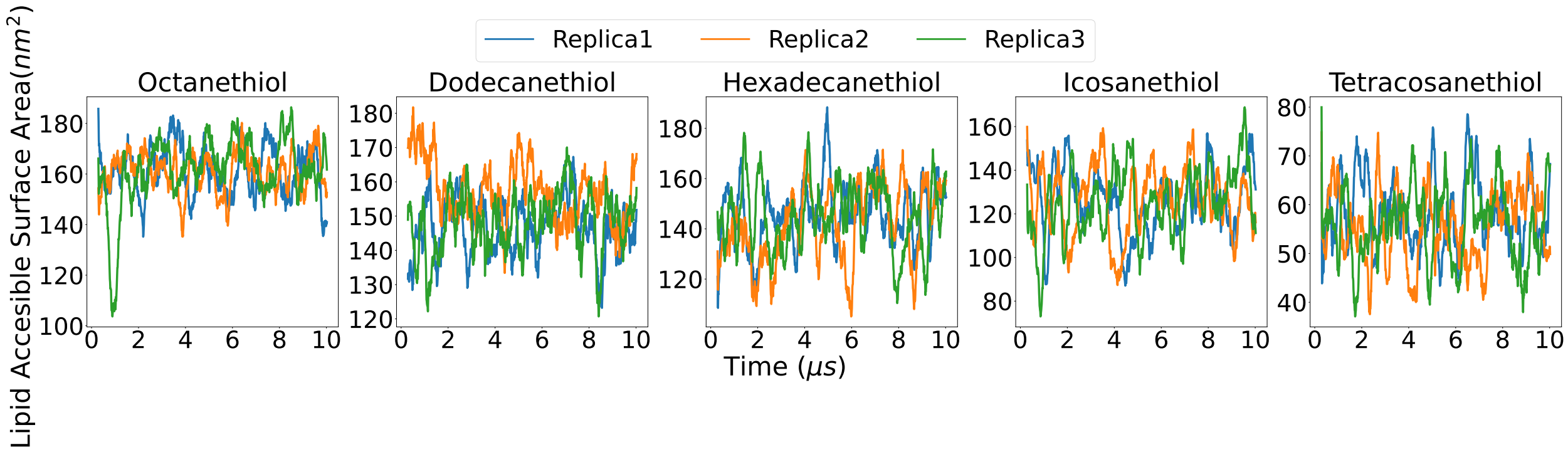

Figure S23: **Convergence of Solvent Accessible Surface Area (SASA) for nanoparticles with N0 bead cores.** Time series of the lipid-accessible surface area for nanoparticles with ligand lengths ranging from C8 (octanethiol) to C24 (tetracosanthiol). Each panel displays N=3 independent replicas (colored lines); data are presented as a 300-ns moving average. The consistency across replicas indicates that the systems have reached a stationary state with respect to the SASA.

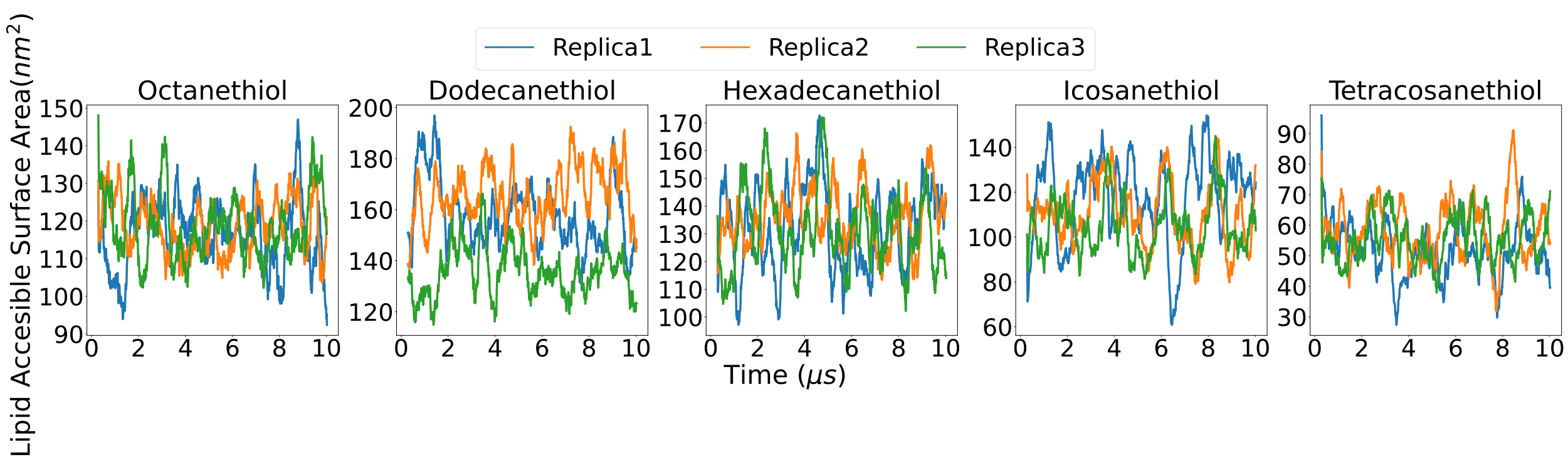

Figure S24: **Convergence of Solvent Accessible Surface Area (SASA) for nanoparticles with P1 bead cores.** Time series of the lipid-accessible surface area for nanoparticles with ligand lengths ranging from C8 (octanethiol) to C24 (tetracosanthiol). Each panel displays N=3 independent replicas (colored lines); data are presented as a 300-ns moving average. The consistency across replicas indicates that the systems have reached a stationary state with respect to the SASA.

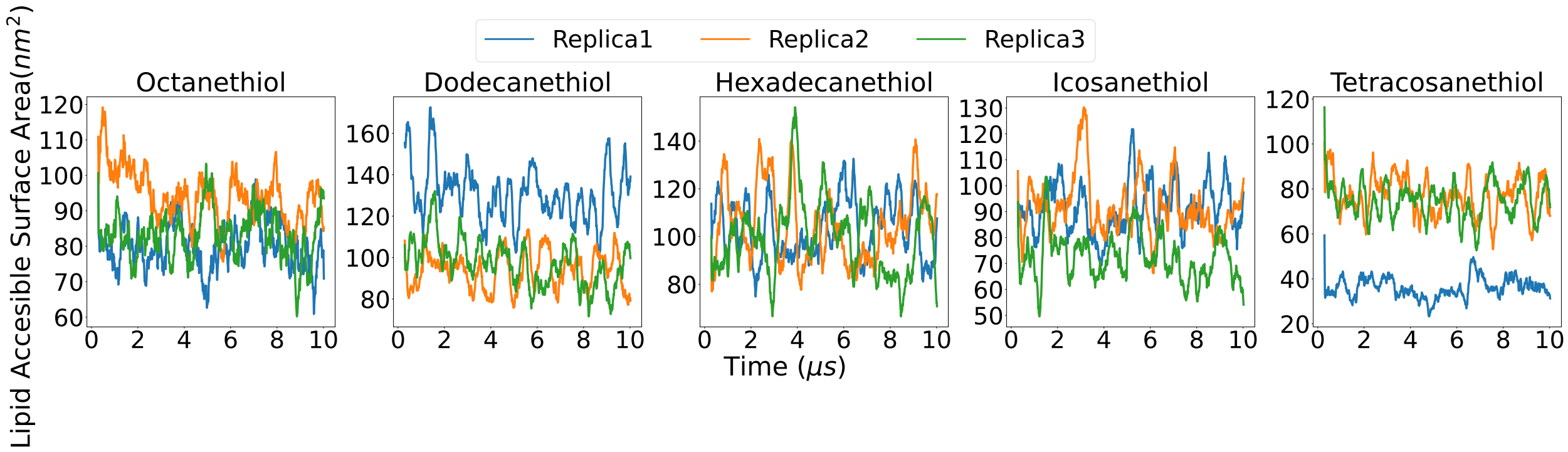

Figure S25: **Convergence of Solvent Accessible Surface Area (SASA) for nanoparticles with P5 bead cores.** Time series of the lipid-accessible surface area for nanoparticles with ligand lengths ranging from C8 (octanethiol) to C24 (tetracosanthiol). Each panel displays N=3 independent replicas (colored lines); data are presented as a 300-ns moving average. The consistency across replicas indicates that the systems have reached a stationary state with respect to the SASA.

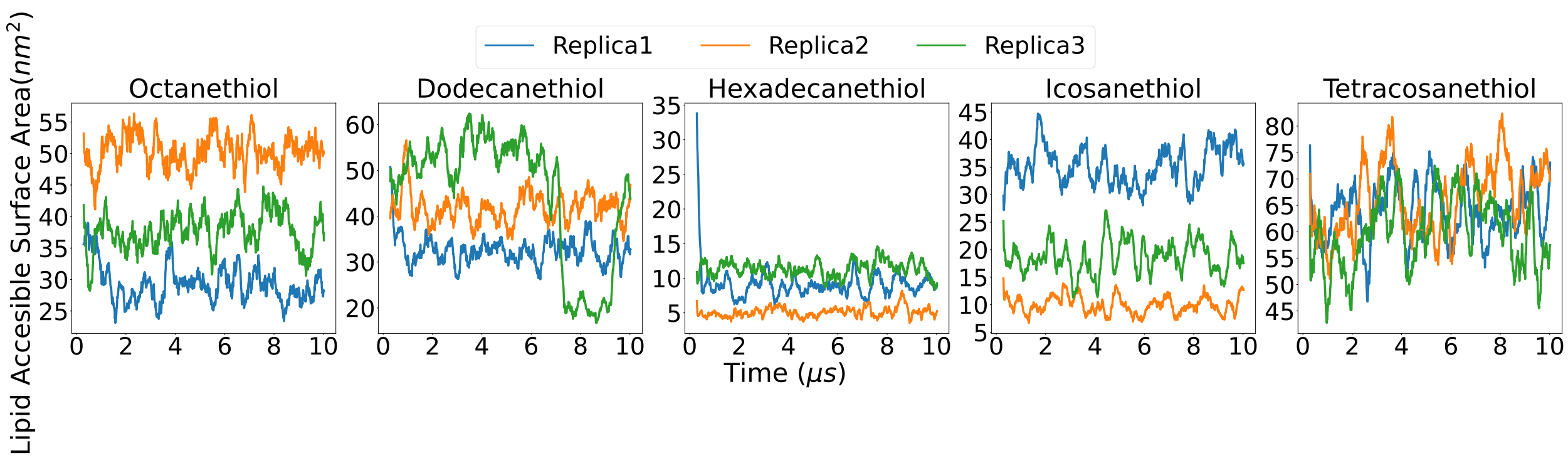

Figure S26: **Convergence of Solvent Accessible Surface Area (SASA) for nanoparticles with SS bead cores.** Time series of the lipid-accessible surface area for nanoparticles with ligand lengths ranging from C8 (octanethiol) to C24 (tetracosanthiol). Each panel displays N=3 independent replicas (colored lines); data are presented as a 300-ns moving average. The consistency across replicas indicates that the systems have reached a stationary state with respect to the SASA.

6 Lipid contacts with nanoparticles

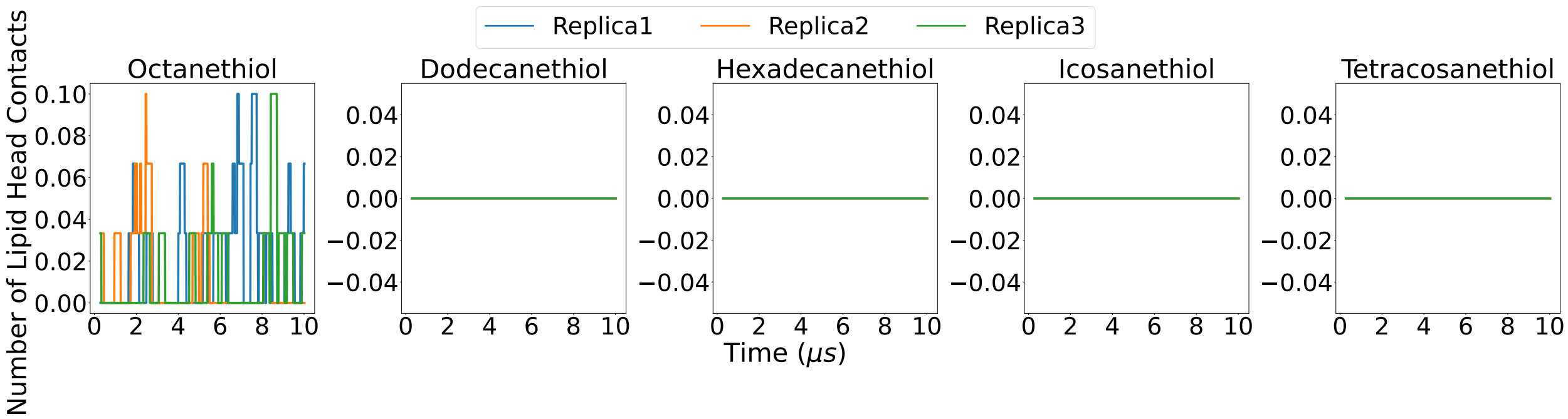

Figure S27: **Convergence of lipid head group contact for nanoparticles with C1 bead cores.** Time series of lipid head group contacts with nanoparticle cores for ligand lengths ranging from C8 (octanethiol) to C24 (tetracosanthiol). Lipid is considered to contact the nanoparticle core if it is within 8 Å of the surface. Each panel displays N=3 independent replicas (colored lines); data are presented as a 300-ns moving average. The consistency across replicas indicates that the systems have reached a stationary state with respect to lipid head-group contacts.

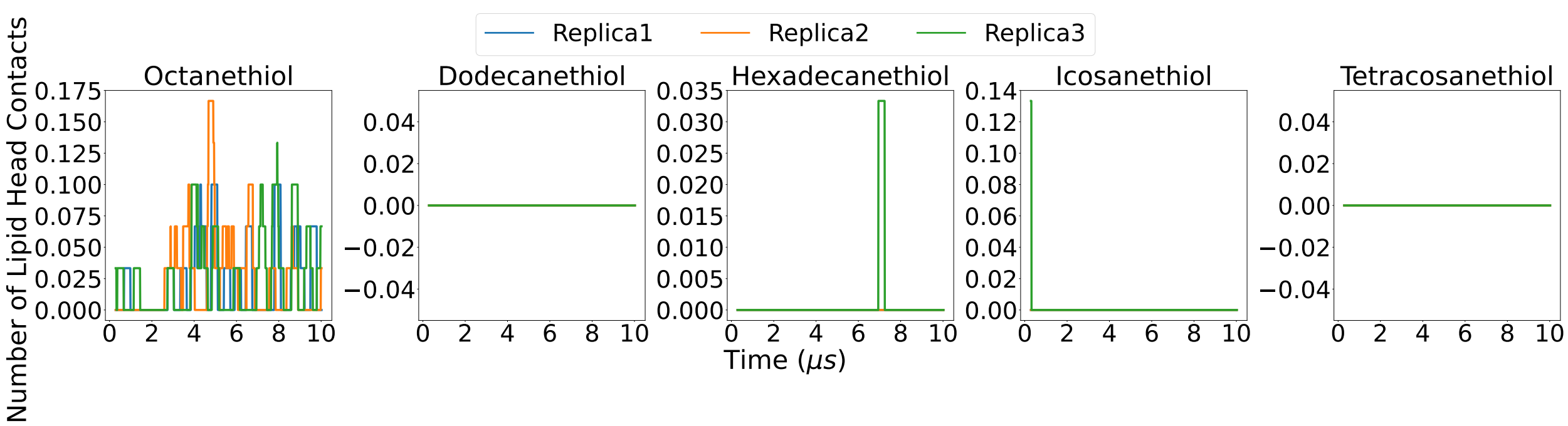

Figure S28: **Convergence of lipid head group contact for nanoparticles with C5 bead cores.** Time series of lipid head group contacts with nanoparticle cores for ligand lengths ranging from C8 (octanethiol) to C24 (tetracosanthiol). Lipid is considered to contact the nanoparticle core if it is within 8 Å of the surface. Each panel displays N=3 independent replicas (colored lines); data are presented as a 300-ns moving average. The consistency across replicas indicates that the systems have reached a stationary state with respect to lipid head-group contacts.

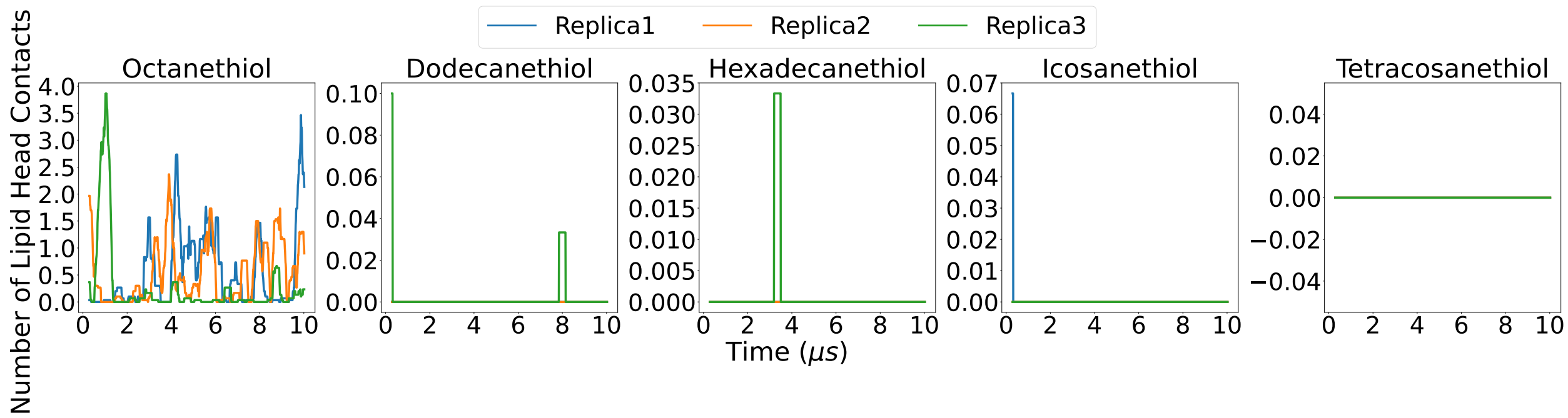

Figure S29: **Convergence of lipid head group contact for nanoparticles with N0 bead cores.** Time series of lipid head group contacts with nanoparticle cores for ligand lengths ranging from C8 (octanethiol) to C24 (tetracosanthiol). Lipid is considered to contact the nanoparticle core if it is within 8 Å of the surface. Each panel displays N=3 independent replicas (colored lines); data are presented as a 300-ns moving average. The consistency across replicas indicates that the systems have reached a stationary state with respect to lipid head-group contacts.

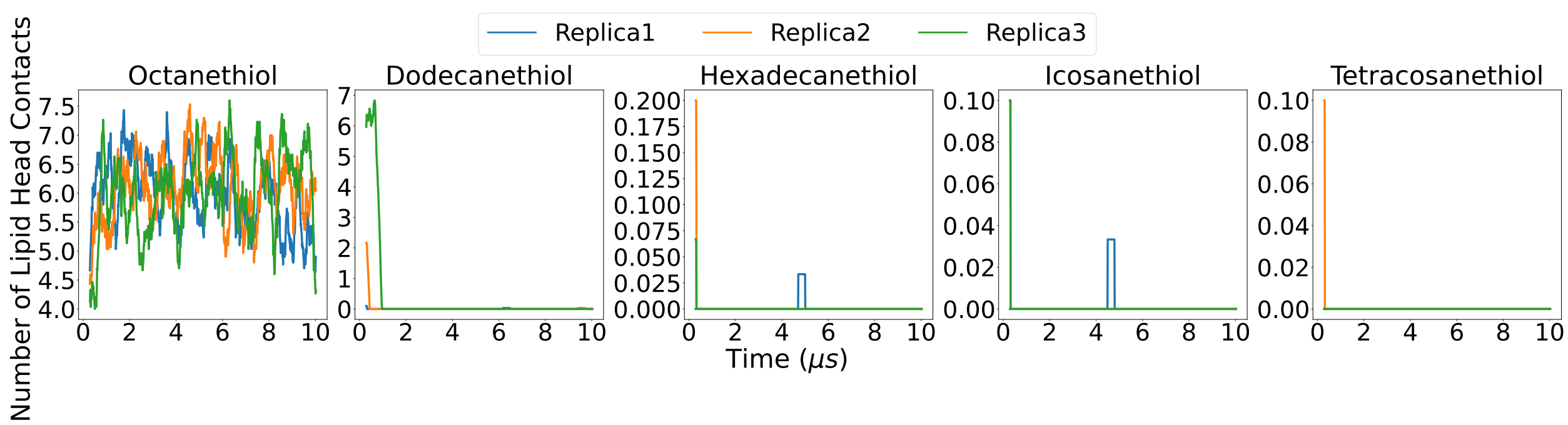

Figure S30: **Convergence of lipid head group contact for nanoparticles with P1 bead cores.** Time series of lipid head group contacts with nanoparticle cores for ligand lengths ranging from C8 (octanethiol) to C24 (tetracosanthiol). Lipid is considered to contact the nanoparticle core if it is within 8 Å of the surface. Each panel displays N=3 independent replicas (colored lines); data are presented as a 300-ns moving average. The consistency across replicas indicates that the systems have reached a stationary state with respect to lipid head-group contacts.

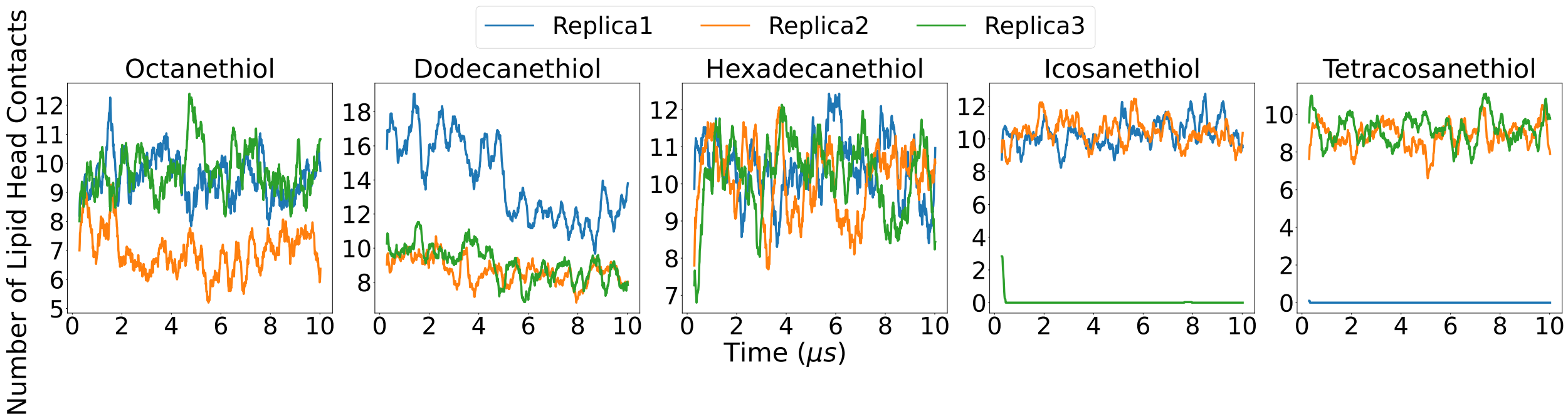

Figure S31: **Convergence of lipid head group contact for nanoparticles with P5 bead cores.** Time series of lipid head group contacts with nanoparticle cores for ligand lengths ranging from C8 (octanethiol) to C24 (tetracosanthiol). Lipid is considered to contact the nanoparticle core if it is within 8 Å of the surface. Each panel displays N=3 independent replicas (colored lines); data are presented as a 300-ns moving average. The consistency across replicas indicates that the systems have reached a stationary state with respect to lipid head-group contacts.

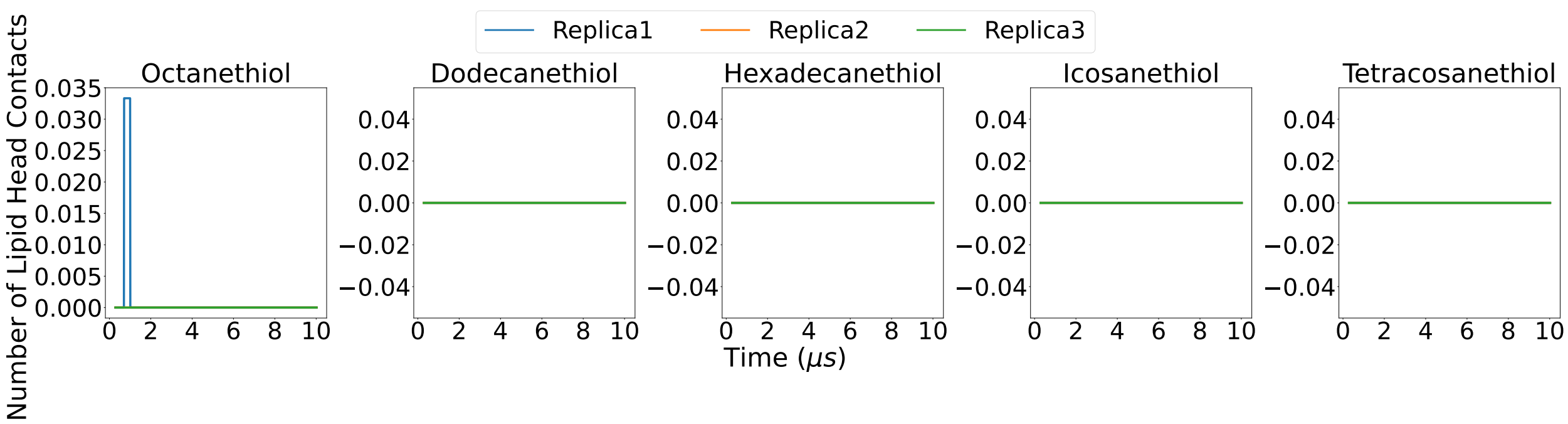

Figure S32: **Convergence of lipid head group contact for nanoparticles with SS bead cores.** Time series of lipid head group contacts with nanoparticle cores for ligand lengths ranging from C8 (octanethiol) to C24 (tetracosanthiol). Lipid is considered to contact the nanoparticle core if it is within 8 Å of the surface. Each panel displays N=3 independent replicas (colored lines); data are presented as a 300-ns moving average. The consistency across replicas indicates that the systems have reached a stationary state with respect to lipid head-group contacts.

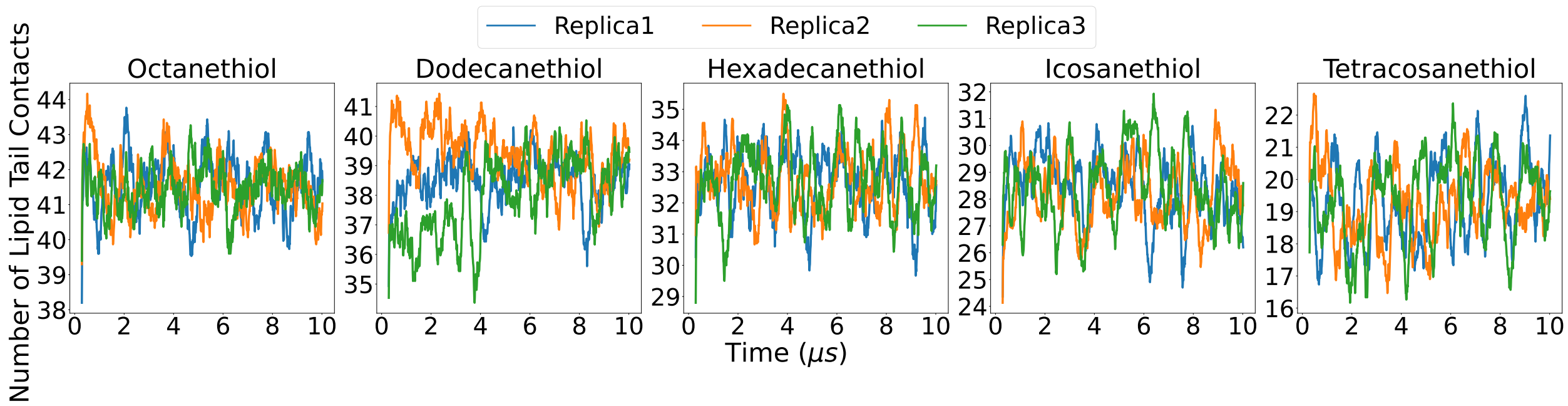

Figure S33: **Convergence of lipid tail group contact for nanoparticles with C1 bead cores.** Time series of lipid head group contacts with nanoparticle cores for ligand lengths ranging from C8 (octanethiol) to C24 (tetracosanthiol). Lipid is considered to contact the nanoparticle core if it is within 8 Å of the surface. Each panel displays N=3 independent replicas (colored lines); data are presented as a 300-ns moving average. The consistency across replicas indicates that the systems have reached a stationary state with respect to lipid tail-group contacts.

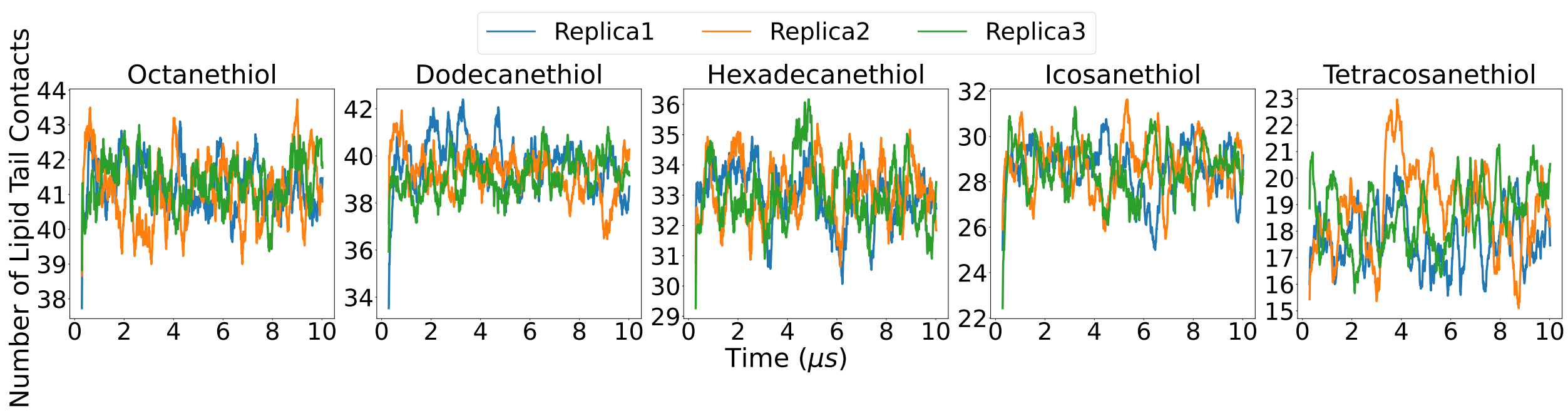

Figure S34: **Convergence of lipid tail group contact for nanoparticles with C5 bead cores.** Time series of lipid head group contacts with nanoparticle cores for ligand lengths ranging from C8 (octanethiol) to C24 (tetracosanthiol). Lipid is considered to contact the nanoparticle core if it is within 8 Å of the surface. Each panel displays N=3 independent replicas (colored lines); data are presented as a 300-ns moving average. The consistency across replicas indicates that the systems have reached a stationary state with respect to lipid tail-group contacts.

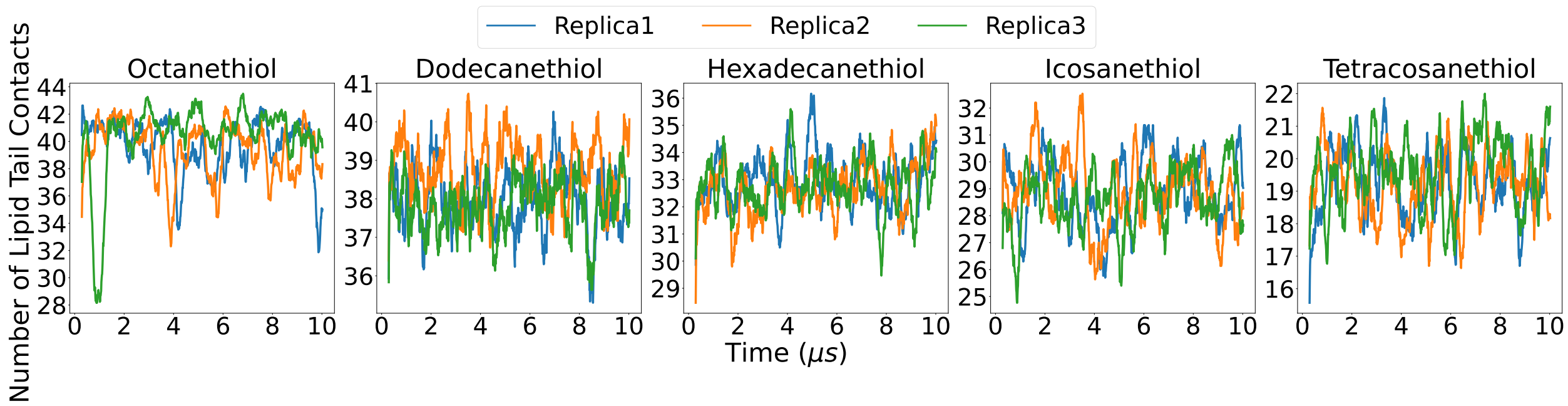

Figure S35: **Convergence of lipid tail group contact for nanoparticles with N0 bead cores.** Time series of lipid head group contacts with nanoparticle cores for ligand lengths ranging from C8 (octanethiol) to C24 (tetracosanthiol). Lipid is considered to contact the nanoparticle core if it is within 8 Å of the surface. Each panel displays N=3 independent replicas (colored lines); data are presented as a 300-ns moving average. The consistency across replicas indicates that the systems have reached a stationary state with respect to lipid tail-group contacts.

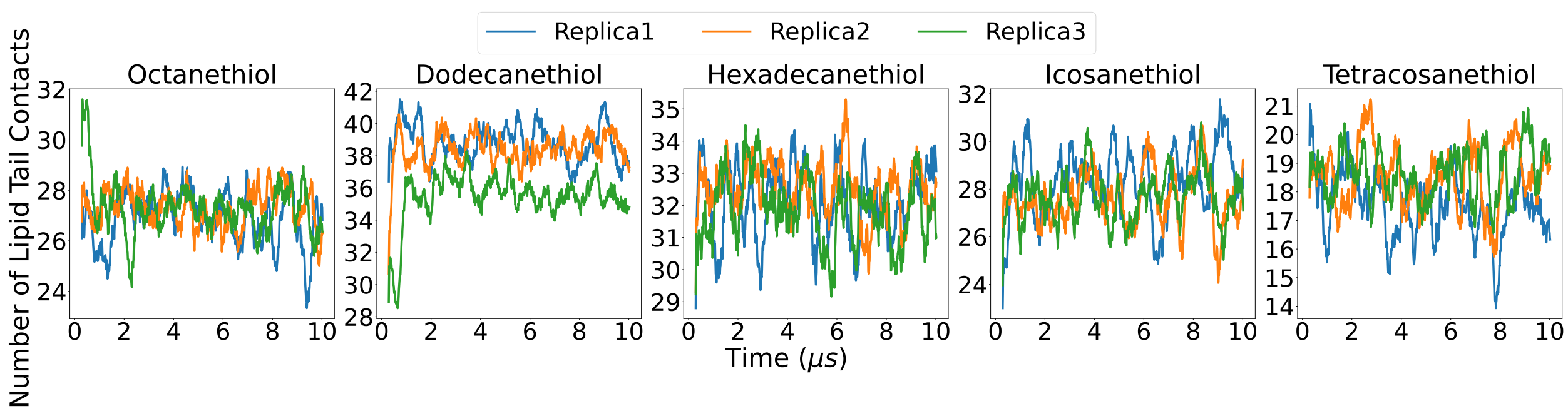

Figure S36: **Convergence of lipid tail group contact for nanoparticles with P1 bead cores.** Time series of lipid head group contacts with nanoparticle cores for ligand lengths ranging from C8 (octanethiol) to C24 (tetracosanthiol). Lipid is considered to contact the nanoparticle core if it is within 8 Å of the surface. Each panel displays N=3 independent replicas (colored lines); data are presented as a 300-ns moving average. The consistency across replicas indicates that the systems have reached a stationary state with respect to lipid tail-group contacts.

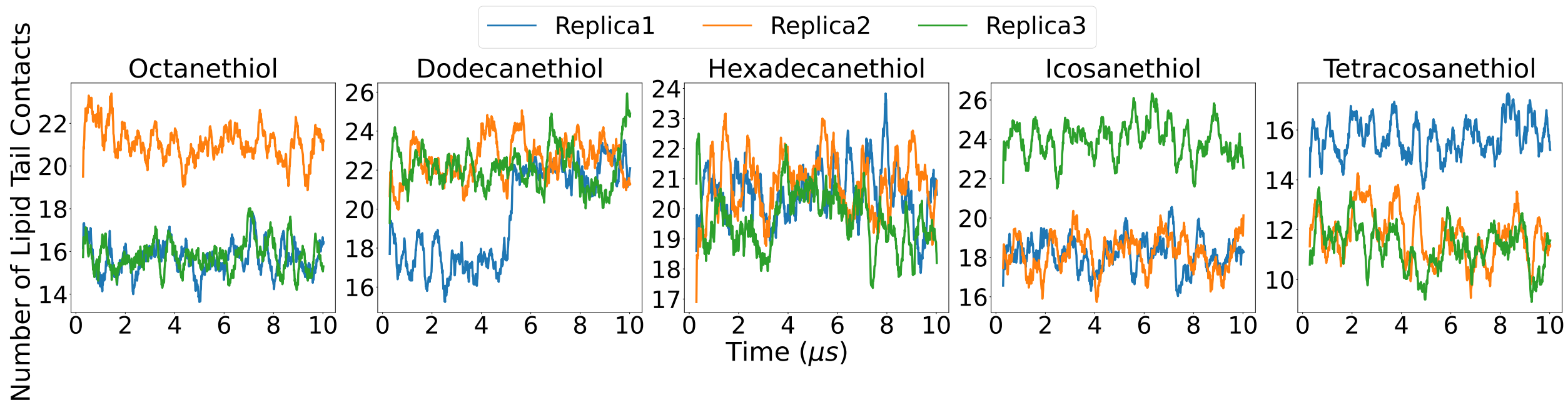

Figure S37: **Convergence of lipid tail group contact for nanoparticles with P5 bead cores.** Time series of lipid head group contacts with nanoparticle cores for ligand lengths ranging from C8 (octanethiol) to C24 (tetracosanthiol). Lipid is considered to contact the nanoparticle core if it is within 8 Å of the surface. Each panel displays N=3 independent replicas (colored lines); data are presented as a 300-ns moving average. The consistency across replicas indicates that the systems have reached a stationary state with respect to lipid tail-group contacts.

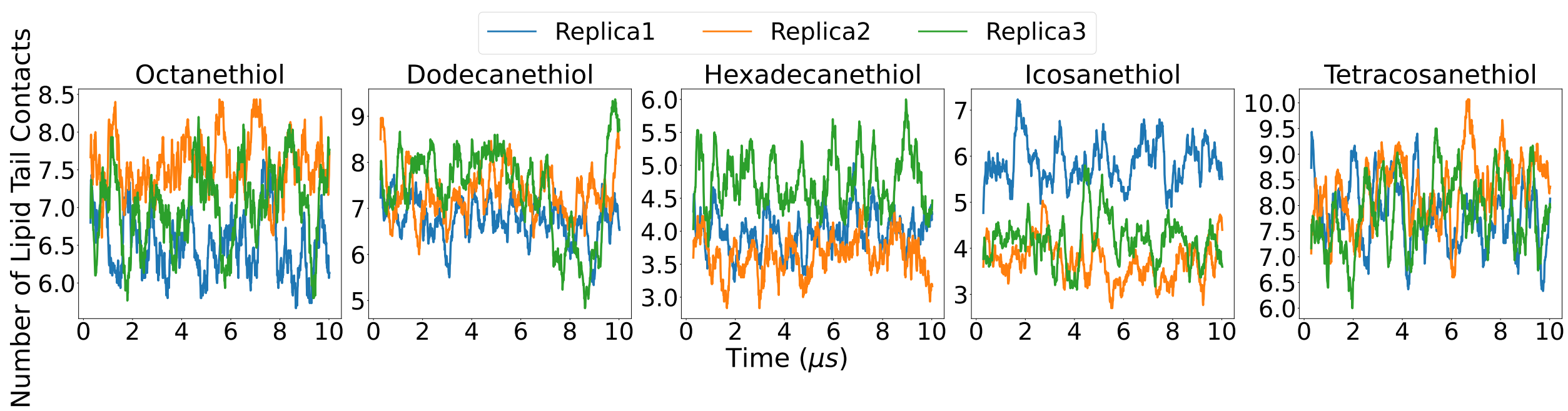

Figure S38: **Convergence of lipid tail group contact for nanoparticles with SS bead cores.** Time series of lipid head group contacts with nanoparticle cores for ligand lengths ranging from C8 (octanethiol) to C24 (tetracosanthiol). Lipid is considered to contact the nanoparticle core if it is within 8 Å of the surface. Each panel displays N=3 independent replicas (colored lines); data are presented as a 300-ns moving average. The consistency across replicas indicates that the systems have reached a stationary state with respect to lipid tail-group contacts.

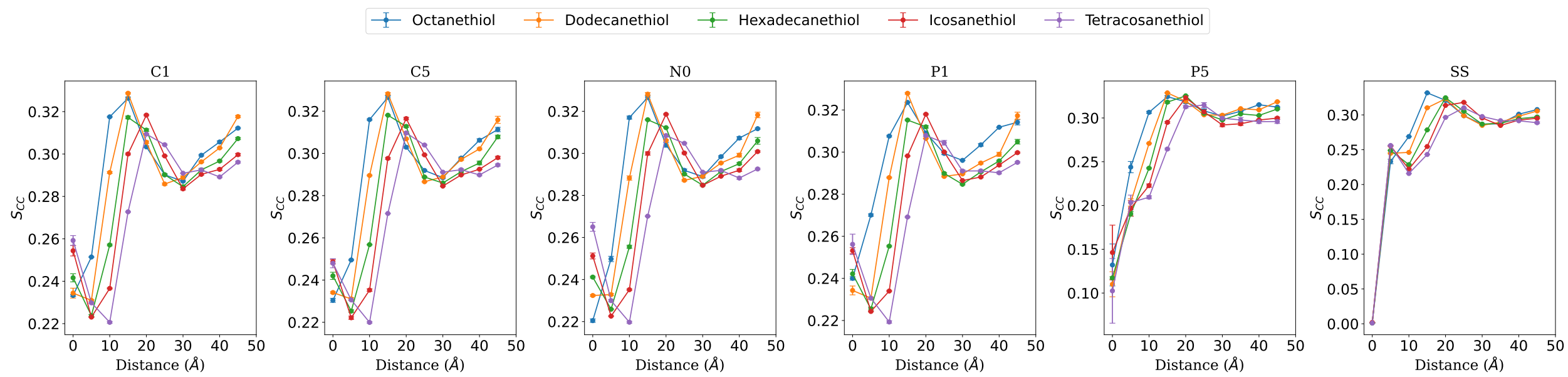

Figure S39: **Phase behavior of POPC membrane with embedded nanoparticles of varying core parameters.** Ensemble-averaged lipid tail order parameter (S_{CC}, measuring tail alignment relative to the bilayer normal) as a function of distance from the nanoparticle surface. Nanoparticles cause a slight decrease in the lipid chain order near the nanoparticle surface, followed by a minor peak around 25 to 30 angstroms, before recovering to bulk values of 0.30 to 0.33 (characteristic of a pure, liquid-disordered Martini 2 POPC bilayer).

7 Methods

Bond and angle parameters follow the harmonic functional forms defined in the GROMACS manual.[1] The harmonic bond potential, $V_b(r_{ij})$, is given by:

$$V_b(r_{ij}) = \frac{1}{2}k^b_{ij}(r_{ij} - b_{ij})^2 \tag{1}$$

where k^b_{ij} represents the bond force constant and b_{ij} the equilibrium distance. Similarly, the harmonic angle potential, $V_\theta(\theta_{ijk})$, is expressed as:

$$V_\theta(\theta_{ijk}) = \frac{1}{2}k^\theta_{ijk}(\theta_{ijk} - \theta^0_{ijk})^2 \tag{2}$$

where k^θ_{ijk} is the angular force constant and θ^0_{ijk} is the equilibrium angle. Bond and angle parameters for the ligands were adapted from the parameterization by Lin et al.[2] Non-bonded interactions between Martini beads are described by the Lennard-Jones (LJ) 12-6 potential:[1]

$$V_{LJ}(r_{ij}) = 4\epsilon_{ij}\left[\left(\frac{\sigma_{ij}}{r_{ij}}\right)^{12} - \left(\frac{\sigma_{ij}}{r_{ij}}\right)^{6}\right] \tag{3}$$

For the soft-sphere (SS) core model, the attractive term was eliminated by setting the coefficient of the $(1/r)^6$ term to zero, resulting in a purely repulsive r^{-12} potential.

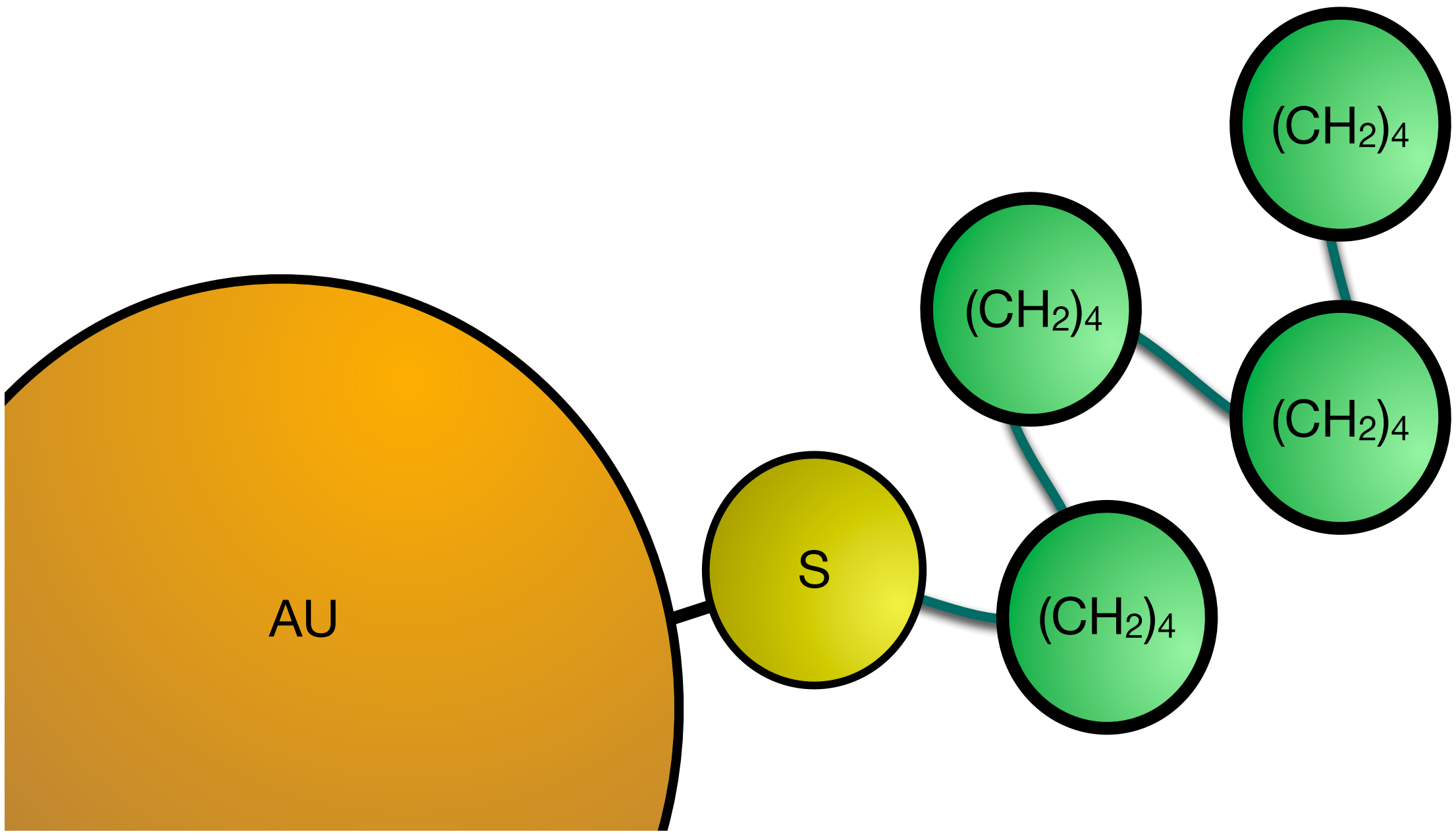

Figure S40: **Schematic representation of the coarse-grained ligand bond topology used for nanoparticle parameterization.**

Table S1: Harmonic bond parameters used for coarse-grained nanoparticle ligands.

Building Block	b_{ij}(nm)	$k_{ij}(\frac{kJ}{mol})$
S-$(CH_2)_4$	0.445	1250
$(CH_2)_4$-$(CH_2)_4$	0.470	1250

Table S2: Harmonic angle parameters used for coarse-grained nanoparticle ligands.

Building Block	θ^0_{ijk}	$k^{\theta}_{ijk}(\frac{kJ}{mol})$
S-$(CH_2)_4$-$(CH_2)_4$	180	25
$(CH_2)_4$-$(CH_2)_4$-$(CH_2)_4$	180	25

References

(1) Abraham, M. et al. GROMACS 2026.1 Manual. **2026**,

(2) Lin, J.-Q.; Zhang, H.-W.; Chen, Z.; Zheng, Y.-G.; Zhang, Z.-Q.; Ye, H.-F. Simulation Study of Aggregations of Monolayer-Protected Gold Nanoparticles in Solvents. *J. Phys. Chem. C* **2011**, *115*, 18991–18998.
